# Comparison of Phenolic Compounds, Carotenoids, Amino Acid Composition, In Vitro Antioxidant and Anti-Diabetic Activities in the Leaves of Seven Cowpea (*Vigna unguiculata*) Cultivars

**DOI:** 10.3390/foods9091285

**Published:** 2020-09-12

**Authors:** Mapula R. Moloto, Anh Dao T. Phan, Jerry L. Shai, Yasmina Sultanbawa, Dharini Sivakumar

**Affiliations:** 1 Phytochemical Food Network Research Group, Department of Crop Sciences, Tshwane University of Technology, Pretoria West 0001, South Africa; mapularebahlotsem@gmail.com; 2 ARC Industrial Transformation Training Centre for Uniquely Australian Foods, Queensland Alliance for Agriculture and Food Innovation, The University of Queensland, St. Lucia, QLD 4108, Australia; a.phan1@uq.edu.au (A.D.T.P.); y.sultanbawa@uq.edu.au (Y.S.); 3 Department of Biomedical Sciences, Tshwane University of Technology, Arcadia, Pretoria 0001, South Africa; ShaiLJ@tut.ac.za

**Keywords:** leafy vegetables, polyphenols, anti-diabetic enzymes, protocatechuic acid-*O*-glucoside, lutein, phenylalanine, antioxidant capacity, regulation of glucose transporter

## Abstract

Cowpea is a well-known nutrition rich African leafy vegetable that has potential to sustain food and nutrition insecurity in sub-Saharan Africa. Consumption of cowpea legumes is associated with reduced risk of type 2 diabetes mellitus. Therefore, the present study was designed to evaluate the (i) variation in phenolic metabolites in seven cowpea cultivars (VOP1, VOP2, VOP3, VOP4, VOP5, VOP7, and VOP8 using UHPLC coupled with high resolution Q-TOF-MS technique, (ii) in vitro antioxidant activity using ferric reducing/antioxidant capacity (FRAP) assay (iii) in vitro anti-diabetic effects and (iv) composition of carotenoids and amino acids of theses cowpea cultivars. The results of this study demonstrated that gentisic acid 5-*O*-glucoside, quercetin 3-(2G-xylosylrutinoside) and Quercetin 3-glucosyl-(1->2)-galactoside were highest in VOP1 VOP4 and VOP5, respectively. High inhibition (>50%) of α-glucosidase and α-amylase activities was shown by the leaf extracts (50 and 25 mg/mL) of VOP1 and VOP4. Cowpea cultivars VOP1 and VOP4 demonstrated the highest gene expression levels of regulation of glucose transporter *GLUT4* in C2C12 skeletal muscle cells, similar to insulin. A positive correlation exited between the phenolic components and the inhibitory effect of antidiabetic enzymes and FRAP activity. Cytotoxic effect was not detected in vitro in any cowpea cultivar. Lutein (124.6 mg/100 g) and all-trans-beta-carotene (92.6 mg/100 g) levels were highest in VOP2 and VOP1, respectively. Cowpea cultivars VOP3 and VOP4 showed potential to fulfil the daily requirements of essential amino acids. Thus, based on this information, cowpea (leaves) genotypes/cultivars can be selected and propagated for the further development of supplementary foods or functional food ingredients.

## 1. Introduction

Cowpea (family Fabaceae) is one of the indigenous leafy vegetables that contributes significantly to household food and nutritional security and societal health, as well as adding variety to cereal-based staple diets in the Southern African region [1]. Cowpea is drought tolerant and well adapted for warm weather conditions. Indigenous fruits and vegetables can be considered as an affordable strategy in diet diversification and the eradication of hidden hunger [2]. In addition, the inclusion of cowpea leaves in African cuisine will add more nutritional value to the consumers as they are a rich source of protein, functional compounds (amino acids, polyphenols, and carotenoids), vitamins (provitamin A, folate, thiamin, riboflavin, and vitamin C) and minerals (calcium, phosphorus, and iron) [3]; the protein content of cowpea leaves ranges from 21.5 to 43.7% [4]. The protein content in cowpea leaves are relatively comparable with other protein-rich leafy vegetables such as spinach (38.2%), Brussels sprouts (34.1%), kale (36.8%) and mustard greens (34%) [5].

Cowpea leaves consist of protein building blocks, essential amino acids, such as isoleucine, leucine, lysine, methionine, phenylalanine, threonine, tryptophan, valine and histidine [4], and the non-essential amino acids tyrosine, aspartate, glutamate, glycine, alanine, cysteine, serine and proline [4]. Research findings of Van Jaarsveld et al. [6] stated that 3/4 cup (90 g) of cowpea leaves fulfil ≥75% of recommended dietary allowance (RDA) for vitamin A (700–900 µg/day for adults [7]; and 25–50% RDA for Fe (10 mg/day) for children (4–8 years) [8].

Consumption of cowpea legumes is associated with reduced risk of type 2 diabetes mellitus and obesity [3]; moreover, the dietary phenolic compounds demonstrated inactivation of carbohydrate digestive enzymes, α-amylase and β-glucosidase, and activated appropriate antihypoglycemic agents [9]. The aforementioned enzymes play a vital role in controlling blood glucose levels and obesity due to their ability to reduce the re-absorption of glucose in the intestine. Inclusion of higher dietary fibre and low glycaemic index foods in daily meals has been proved to reduce blood glucose levels, thereby reducing the risk of type 2 diabetes mellitus [9]. Indigenous edible plant extracts demonstrated anti-diabetic effects [10], such as insulin (diabetic drug), by executing the vital regulatory mechanism transporting the glucose uptake into skeletal muscle and adipose tissue by facilitating glucose transporter protein type-4 (GLUT4), playing a major role in the management of type 2 diabetes [11]. GLUT4 therefore plays a vital role in glucose homeostasis of skeletal muscle cells and the removal of glucose from blood circulation [11].

The number of people affected with type 2 diabetes in Africa was projected to increase to 41.5 million in 2035, and it will be more prevalent in people who are between the ages of 40–59 [12]. Another approach to tackle type 2 diabetes and obesity is formulating supplementary foods using indigenous plant ingredients based on their nutritional values and phytochemical profiles. Implementing supplementary feeding programmes would be an affordable strategy and would result in lowering postprandial glycaemia at least partly by promoting skeletal muscle glucose uptake and intensifying the metabolism.

Chemometric analysis is widely used in metabolomics analysis for characterisation, and assessment of the divisiveness in the overall bioactive metabolites of functional foods [13]. In addition, it is essential to build up a phytochemical database for bioactive compounds in foods that can link to the chemical properties associated with nutritional and nutraceutical effects [13]. Therefore, this study aimed to (i) investigate the different phenolic metabolites in cowpea accessions using UHPLC coupled with high resolution Q-TOF-MS technique and a chemometric analysis approach, (ii) characterise and quantify the phenolic compounds carotenoids and amino acids, (iii) determine the in vitro antioxidant capacity and anti-diabetic activity, and (iv) understand the molecular basis for ‘insulin like’ activity of the polyphenols extracted from different African grown cowpea leaf cultivars, on the regulation of glucose transporter *GLUT4.* This study will lead in identifying cowpea cultivars, for the development of supplementary foods that are a rich source of amino acids, carotenoids, phenolic compounds and antioxidant properties, and relate to the dietary roles of cowpea (leaf) functional compounds in type 2 diabetes management. This approach will significantly benefit the consumers belonging to the vulnerable groups and the food manufacturers.

## 2. Materials and Methods

### 2.1. Chemicals

Polyphenols (≥95% purity), including chlorogenic acid, catechin, luteolin, epicatechin and rutin, were purchased from Sigma-Aldrich (Johannesburg, South Africa). Carotenoids (analytical standards), including violaxanthin, lutein, zeaxanthin and all-trans-beta-carotene, were sourced from Sigma-Aldrich (Sydney, NSW, Australia). All the other chemicals and solvents (HPLC grade) were purchased from Merck (Darmstadt, Germany) or Sigma-Aldrich (Johannesburg, South Africa).

### 2.2. Plant Material

**Figure 1:**
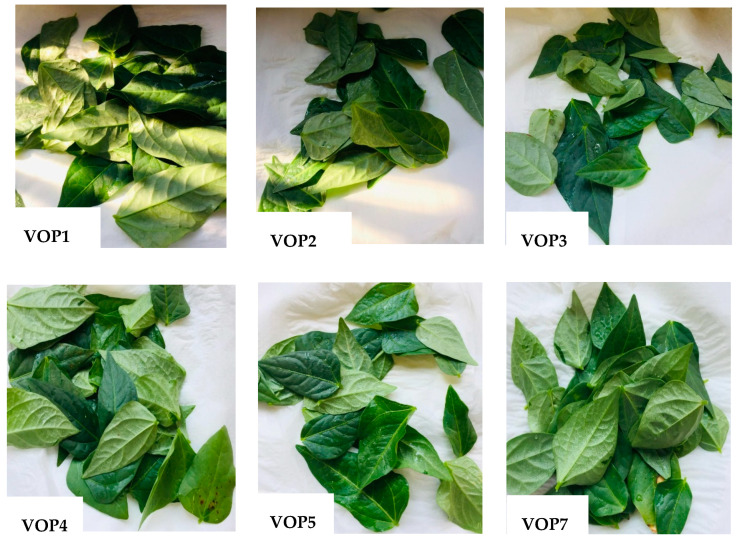

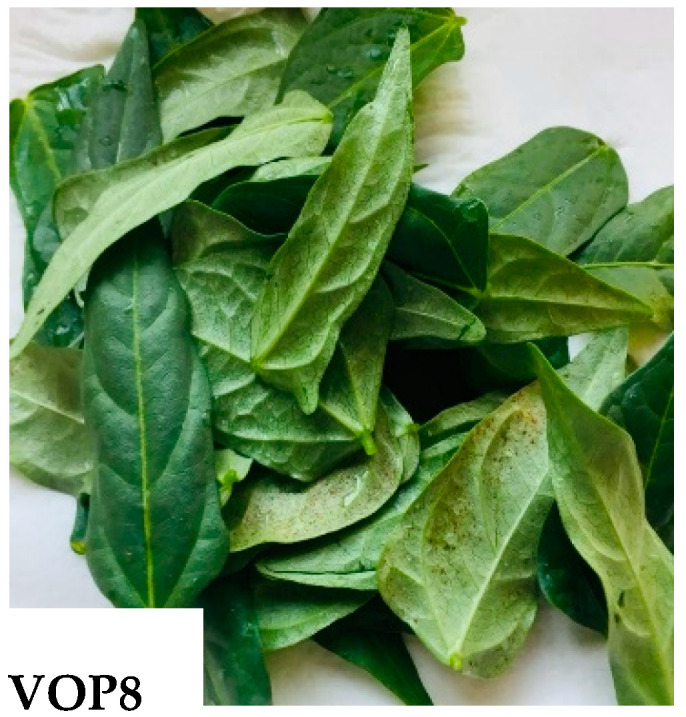
Different cowpea cultivars used in this study.

Seeds of seven cowpea (*Vigna unguiculata* L. Walp) cultivars (VOP1, VOP2, VOP3, VOP4, VOP5, VOP7, VOP8) (Figure 1) planted in Southern African region were obtained from Dr. Abe Gerrano and Ms. Lindiwe Khoza from the Agricultural Research Council’s (Pretoria, South Africa). Cowpea plants were propagated at the experimental plot at the Tshwane University of Technology (25°43′53.55″S, 28°09′40.38″E, on 1230 m.a.s.l) during the summer of 2018, and the average temperature ranged from 16 to 30 °C. The experimental unit was arranged in a completely randomised design with five replications for each cowpea cultivar and the seeds planted in pots. The irrigation (100 mL/day) was kept to a minimum, as its production was recommended in dry lands. The leaves were harvested at 8-leaf stage, reached after 60 to 95 days of planting. Leaves (1.5 kg) that were free from decay, damage or soil particles were harvested and rinsed in tap water, then snap frozen in liquid nitrogen and subsequently stored at −80 °C for biochemical analysis. Another portion of leaves (150 mg) was freeze-dried (−85 °C, LyoQuest −55/Telstar, Shanghai, China) and ground into fine powder for carotenoid and total protein content and amino acid analysis.

### 2.3. Predominant Phenolic Metabolic Profile

Phenolic compounds were extracted from cowpea leaves using the method described by Ndou et al. [14] and Managa et al. [15]. Cowpea snap frozen leaf samples (50 mg) were extracted in ethanol/water solution (70:30, *v*/*v*), ultrasonicated for 30 min then centrifuged (Hermle Z326k, Hermle Labortechnik GmbH, Wehingen, Germany) at 1000× *g* for 20 min at 4 °C. The supernatants were collected and filtered through a 0.22-µm polytetrafluorethylene filter prior to UPLC-QTOF/MS analysis.

Peak identities and quantification of predominant polyphenol metabolites were carried out using an UPLC-QTOF/MS system (Waters, Milford, MA, USA) equipped with a Quadrupole 120 time-of-flight (QTOF) mass spectrometer. The chromatographic conditions were performed as per Ndou et al. [14] and Managa et al. [15]. Due to the unavailability of commercial standards, these were semi-quantitatively measured against calibration curves prepared using chlorogenic acid, catechin, luteolin, epicatechin and rutin. Data processing using TargetLynx software was conducted as described previously [14,15]. The differences between the phenolic metabolic profiles of the different cowpea cultivars were analysed using an unsupervised Principal Component Analysis (PCA) approach using the data generated by the UPLC–Q-TOF/MS analysis. PCA was performed to reduce the number of variables in the data matrix in order to select the most discriminating cowpea cultivars as stated by Ndou et al. [14]. Therefore, the UPLC data were exported as an mzXML file and aligned by Marker Lynx 4.1 in the Apex Trac ™ tool and imported into SIMCA-P + 12.0 and fir the PCA analysis. However, to explain the differences between the cultivar groups and to identify the potential characteristic markers (metabolites) responsible for discrimination between the cowpea cultivars, supervised Orthogonal Projections to Latent Structures Discriminant Analysis (OPLS-DA) was performed.

### 2.4. Trolox Equivalent Antioxidant Capacity (TEAC) FRAP Assay

FRAP assay was carried out according to Mpai et al. [16], using a micro-plate reader (CLARIOstar Plus BMG Labtec, Lasec, Cape Town, South Africa) and snap frozen cowpea leaf samples (0.2 g). Briefly, 15 µL aliquot of leaf extract and 220 µL of FRAP reagent solution were added to the wells. The absorbance was read at 593 nm and the standard curve of Trolox was constructed to calculate the reducing antioxidant capacity was expressed as µmol TEAC/100 g.

### 2.5. Antidiabetic Activity

#### 2.5.1. α-Glucosidase Inhibition Assay

The α-glucosidase inhibitory activity was measured according to the method described by Sagbo et al. [17], using a micro-plate reader (CLARIOstar Plus BMG Labtec, Lasec, Cape Town, South Africa). Briefly, 5 µL of the leaf extract (mentioned in Materials and methods Section 2.3) of cowpea cultivars VOP1 and VOP4 (50, 25 and 5 mg/µL) was mixed with 20 µL of 50 µg/mL α-glucosidase solution into a well, then 60 µL of 67 mM potassium phosphate buffer (pH 6.8) was added to the mixture and incubated for 5 min at 35 °C. Subsequently, 10 µL of 10 mM ρ-nitrophenyl-α-*D*-glucoside solution (PNPGLUC) was added and the incubation was extended for an additional 20 min at 35 °C, followed by adding 25 µL of 100 mM Na_2_CO_3_; the absorbance was read at 405 nm. The absorbance was measured for the cowpea leaf extracts, or acarbose, and the blank (samples without α-glucosidase). The enzyme inhibitory activity was calculated according to Sagbo et al. [17] and expressed as the percentage of α-glucosidase inhibition.

#### 2.5.2. α-Amylase Inhibition Assay

The α-amylase inhibition assay was performed according to the method described by Sagbo et al. [17], without any modifications, using a micro-plate reader (CLARIOstar Plus BMG Labtec, Lasec, Cape Town, South Africa) monitored at 580 nm. The enzyme inhibitory activity was expressed as the percentage of α-amylase inhibition.

#### 2.5.3. Gene Expression of GLUT-4

Treatment of cells with the leaf extracts (100 µL) of cowpea cultivars VOP1 and VOP4 (mentioned in Materials and methods 2.3) were performed, according to the method described by Seabi et al. [18], by plating the C2C12 (mouse skeletal muscle, American Type Culture Collection [ATCC], Manassas, VA, USA) cells in 6-well plates at a density of 1 × 10^5^ cells/mL. C2C12 cells, fully differentiated into myotubules, were treated with 100 µL of 50 mg/µL, leaf extract for 3 h prior to the isolation of total RNA. Insulin (10 µg/mL) was included as a control. Cells were detached from the culture plates and centrifuged (Beckman TJ-6, Analytical Instruments Brokers LLC, Golden Valley, MN, USA) at 250× *g* for 5 min. Isolation of total RNA from C2C12 cells was carried out according to Seabi et al. [16], by centrifuging the harvested cells at 250× *g* for 5 min. Thereafter, RNA extraction was performed using an RNA extraction kit (Life Technologies, Johannesburg, South Africa), and the RNA (0.5 µg) was reverse transcribed to cDNA using the cDNA synthesis kit. The cDNA reaction mixture included 10 µL template RNA, 2 µL oligo d (T) primer, 12 µL nuclease-free deionised water, 4 µL 5× reaction buffer, 1 µL RibobLock RNase inhibitor and 1 µL MuLV reverse transcriptase [18]. The reaction was allowed at 42 °C for 60 min; subsequently the temperature was increased to 70 °C for 5 min to terminate the reaction. The polymerase chain reaction (PCR) was performed using a mixture of 2 µL of forward and reverse primers (0.4 µM each), 5 µL of template cDNA, 16 µL of nuclease-free sterile deionised water and 25 µL of 2× ReadyMix (Kapa Biosystems, Wilmington, NC, USA) [18]. The conditions for the PCR reaction were similar to those reported by Seabi et al. [18]. After completion, the resulting products of PCR were analysed on 2% agarose gel electrophoresis (Bio-Rad Laboratories, Sandton, Johannesburg, South Africa), at 75 V for at least 1 h at 25 °C, as described by Seabi et al. [18]. The primers used for the PCR (reverse and complementary) for GLUT 4 are given in Table S1.

### 2.6. Cell Cytotoxicity Using MTT Assay

Cell toxicity was measured by the MTT (3-(4,5-dimethylthiazol-2-yl)-2–5-diphenyltetrazolium bromide, Merck, Johannesburg, South Africa) cytotoxicity assay using C2C12 myoblast cell (mouse skeletal muscle) line, according to a method described by Seabi, et al. [17] without any modifications. Cells were seeded at an initial cell density of 1 × 10^5^ cells/mL in a 96-well cell culture plate. Thereafter, cells were treated with different concentrations (0.25–25 mg/mL) of the different cultivars of cowpea leaf extracts (mentioned in Materials and methods 2.3) and incubated at 37 °C for 24 h. The untreated cells were included as the experimental control; ZnCl_2_ (0.25–2.5 mM) and H_2_O_2_ (0.25–2.5%) were used as positive control. Afterwards, an aliquot of 20 µL of 5 mg/mL MTT (3-(4,5-dimethylthiazol-2-yl)-2,5-diphenyltetrazolium bromide) was added to each well and incubated at 37 °C for an additional 4 h to allow the conversion of MTT to the coloured formazan. Cell cytotoxicity was measured at 570 nm using a microtitre-plate multimode detector (Promega-Glomax Multi-detection system, Madison, WI, USA), using the formula below; the blank well included only the medium.

$$\%\;\mathrm{Viable}\;\mathrm{cells}\;=\frac{*abs\;sample-abs\;blank)}{dbs\;control-abs\;blank}\;\text{×}\;100$$

* *abs*—absorbance.

### 2.7. Carotenoids

Carotenoid extraction was performed according to Djuikwo, et al. [19], with some modifications. Powdered cowpea leaf (100 mg) was homogenised with acetone and 95% ethanol containing 0.1% (*w*/*v*) butylated hydroxytoluene (BHT) in an orbital shaker (RP1812, Paton Scientific, Victor Harbor, SA, Australia) for 10 min. The samples were saponified at 25 °C for 30 min in KOH (20% in methanol, *w*/*v*) while shaking at 100 rpm. Afterwards, hexane/dichloromethane mixture (70:30, *v*/*v*), containing 0.1% BHT, was added to extract carotenoid compounds into the upper phase. NaCl (10%, *w*/*v*) was added for phase separation, thereafter, centrifuged at 3900× *g* for 5 min at 25 °C (Eppendorf 5804, Lasec Pty, Midrand, South Africa). The upper layer was collected, combined and evaporated under nitrogen stream until dry. The crude extract was freshly reconstituted in methanol/MTBE (50:50, *v*/*v*), containing 0.1% BHT, for UHPLC_UV_MSMS analysis.

Carotenoids were analysed using a Dionex Ultimate 3000 UHPLC system (Thermo Fisher Scientific, Waltham, MA, USA) equipped with a Thermo UV detector, scanned at 450 nm, and a Thermo high resolution Q Exactive Quadrupole-Orbitrap mass spectrometer. Compound separation was performed on a YCM C30 column (3.6 × 250 mm, 3.6 µm) (Waters, Milford, MA, USA) maintained at 25 °C, with 0.1% formic acid in methanol (eluent A) and 0.1% formic acid in MTBE (eluent B). The gradient programme of mobile phase A was as follows: (0 min, 80%), (20 min, 75%), (30 min, 30%), (33 min, 30%), (36 min, 80%), with the flow rate of 0.6 mL/min. Mass spectrometry analysis was operated in positive mode, employing an atmospheric pressure chemical ionisation (APCI). A full MS scan (*m*/*z* 120–1000) was acquired at a resolving power of 70,000 full-width half maximum. For the compounds of interest, an MS/MS scan from *m*/*z* 80 to 650 was selected, with normalised collision energy at 20V. Carotenoids were quantified at 450 nm, using external calibration curves of carotenoid standards stated in Section 2.1. Concentration of carotenoid standards was determined using a Cintra UV-Vis spectrophotometer (GBC Scientific Equipment, Braeside, VIC, Australia), based on specific molar absorption coefficients in solutions as described previously [19].

### 2.8. Amino Acids

Amino acids were quantified according to the method described by Mpai et al. [16]. Freeze-dried frozen cowpea leaves (100 mg) were mixed with 6 N HCl and incubated in an oven at 110 °C for 18 h; thereafter the mixture was cooled, centrifuged, filtered and dried in a speed vac concentrator. It was then derivative by adding 10 µL aliquot of the freshly made undiluted sample containing 20 µL l-Norvaline in 80 µL of the sample to the 20 µL of AccQ-Tag Ultra amino acid kit, vortexed and incubated in the oven at 55 °C for 10 min. The vials were cooled for analysis using a Waters UPLC-PDA system (Waters, Milford, MA, USA). The conditions for UPLC analysis were similar to the method described by Mpai et al. [16]; standard calibration curves were constructed to quantify the amino content and expressed as g/100 g.

### 2.9. Statistical Analysis

The experiments were repeated with two harvests within the season and the data adopted a completely randomised design. As there was no significant variation between the two harvests, the data was pooled together for statistical analysis. For biological activities, three sample replicates per leaf extract concentration per treatment (cowpea cultivars) were analysed, whereas for biochemical analysis a cumulative five replicate samples per treatment, (cowpea cultivars) were included. The data obtained were subjected to analysis of variance (ANOVA) using the statistical programme GenStat version 11.1, statistical data analysis software (Hempstead, England, UK). Treatment means were compared using Fishers protected *t*-test least. Significant difference (LSD) was at the 5% level of significance. Pearson’s correlation coefficients were calculated to determine the strength of the linear relationships between antioxidant capacity, targeted phenolic compounds and antidiabetic enzyme inhibition activity.

## 3. Results and Discussion

### 3.1. Quantification of Targeted Phenolic Metabolites in Cowpea Cultivars

Figure S1 illustrates the total ion chromatograms of phenolic metabolites from the leaves of cowpea cultivars operated in negative ESI-mode using a UPLC–QTOF/MS system. In total, seven compounds that belong to the group of phenolic acid and flavonoid glycosides were identified as main phenolic compounds in cowpea cultivars including gentisic acid 5-*O*-glucoside, p-coumaric acid *O*-glucoside, ferulic acid *O*-glucosid and four quercetin derivatives (quercetin 3-sambubioside-3′-glucoside, quercetin 3-glucosyl-(1->2)-galactoside, quercetin 3-(2G-xylosylrutinoside), and quercetin 3-*O*-rhamnoside 7-*O*-glucosi (Table 1). The MS spectra of these compounds are given in Figure S2A–D.

Peak 1 (gentisic acid 5-*O*-glucoside) showed fragment ion at *m*/*z* 152 due to loss of hexoside [20]. For peak 4 (quercetin 3-sambubioside-3′-glucoside), peak 5 (quercetin 3-glucosyl-(1->2)-galactoside) and peak 7 (quercetin 3-*O*-rhamnoside 7-*O*-glucoside) compounds had a fragment at *m*/*z* 301, which could be attributed to the release of quercetin (aglycone) [20,21,22] (Table 1 and Figure S1D,E,G).

**Figure 2:**
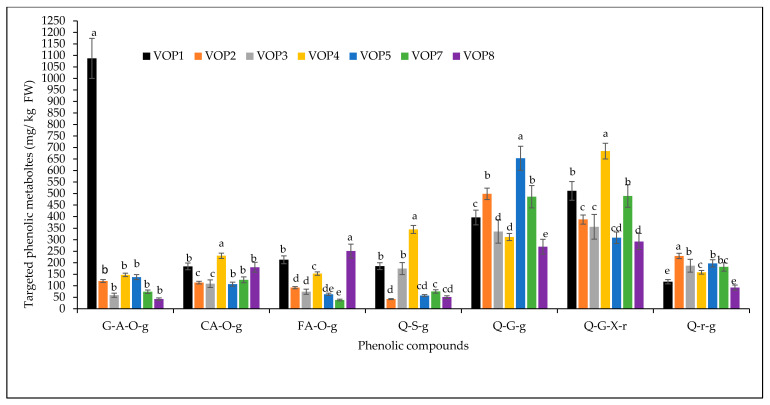
Concentration of targeted phenolic compounds in different cowpea cultivars. FW—fresh weight, Bar with same alphabets are not significantly different between cultivars at *p* < 0.05 for a specific phenolic compound. Data obtained were subjected to analysis of variance (ANOVA) using the statistical programme. Each bar represents the mean and standard deviation (*n* = 3). Gentisic acid 5-*O*-glucoside (G-A-*O*-g); p-Coumaric acid *O*-glucoside (CA-*O*-g); Ferulic acid *O*-glucoside (FA-*O*-g); Quercetin 3-sambubioside-3′-glucoside (Q-S-g); Quercetin 3-glucosyl-(1->2)-galactoside (Q-G-g); Quercetin 3-(2G-xylosylrutinoside) (Q-G-X-r); Quercetin 3-*O*-rhamnoside 7-*O*-glucoside (Q-r-g).

Figure 2 presents the concentrations of seven phenolic metabolites in different cowpea cultivars. Concentration of gentisic acid 5-*O*-glucoside was significantly highest in cowpea cultivars VOP1 (1087 mg/kg), compared to the other cowpea cultivars. The gentisic glucosides were reported in Bitter melon (*Momordica charantia*) [23], and Mutamba (*Guazuma ulmifolia* Lam) fruits [24].

Among the quercetin derivatives, quercetin 3-glucosyl-(1->2)-galactoside and quercetin 3-(2G-xylosylrutinoside) were detected at higher concentrations compared to the other two quercetin derivatives. Quercetin 3-(2G-xylosylrutinoside) was the second dominant phenolic compound that is found in green bean and the significantly highest concentration was found in cowpea cultivar VOP4 (653.4 mg/kg), followed by VOP1 (511.41 mg/kg) and VOP7 (489.47 mg/kg). Quercetin 3-glucosyl-(1->2)-galactoside was obtained in cowpea cultivar VOP5 (653.4 mg/kg), followed by VOP2 (498.6 mg/kg) and VOP7 (486.2 mg/kg); other cowpea cultivars showed significantly lower concentrations of quercetin 3-glucosyl-(1->2)-galactoside. Cowpea cultivar VOP4 contained the highest concentration of quercetin 3-sambubioside-3′-glucoside followed by VOP1 and VOP3. Highest concentrations of quercetin 3-*O*-rhamnoside 7-*O*-glucoside was detected in cowpea cultivar VOP2. Concentrations of coumaric acid *O*-glucoside, and ferulic acid *O*-glucoside were highest in cowpea cultivars VOP4 and VOP8 respectively. Quercetin 3-*O*-xylosylrutinoside or isomers, were reported previously in green beans [20,21,22].

Although the dietary phenolic acids and flavonoids were found in higher concentrations in the cowpea cultivars VOP1 and VOP4, their health benefits depend on their bioavailability [25]. Hollman et al. [26], reported the bioavailability of quercetin glycosides in onions and the pure quercetin rutinoside at 52% and 17%, respectively. Ferulic acid has showed efficient absorption when it exits as free from in tomatoes or beers, but its bioavailability is limited in the ester forms reported in cereals [27]. Further studies on digestive stability, bio accessibility, bioavailability and subsequently bioactivity, both in vitro and *in vivo*, are strongly recommended to get a better understanding of nutritional values of cowpea leaves, an emerging food in the African market. Heat map (Figure 2) demonstrated the quantitative pattern of phenolic metabolites in the leaves of different cowpea cultivars. The pattern and magnitude, relating to the colour intensity (hue) from +2 to −2, with 0 as symmetry, relate to visualisation of response intensities of 19 compounds, including the unidentified compounds.

### 3.2. Multivariate Analysis

**Figure 3:**
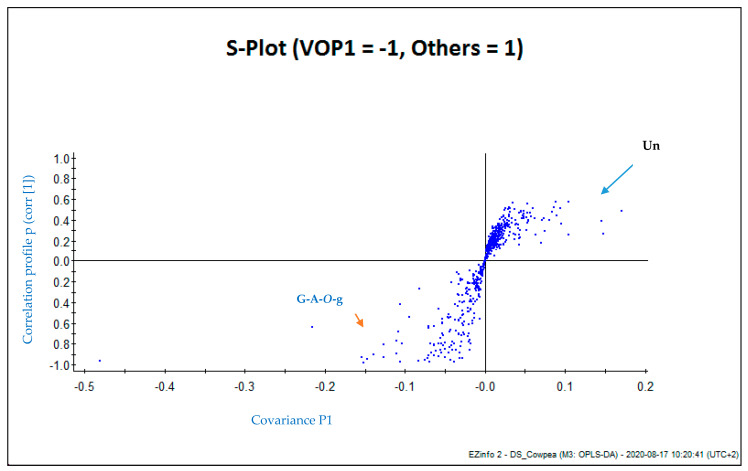
Score plot of orthogonal partial least squares discriminant analysis of UPLC–Q-TOF/MS spectra of the leaves of different cowpea cultivars. Gentisic acid 5-*O*-glucoside (G-A-*O*-g), Un—Unidentified compound.

**Figure 4:**
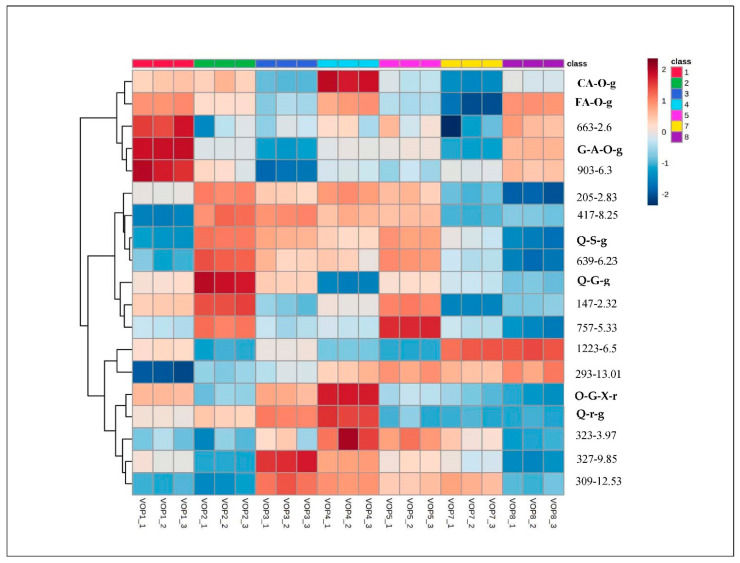
Heat map of nineteen phenolic metabolites (variables) in hierarchical clustering in the leaves of cowpea cultivars. Gentisic acid 5-*O*-glucoside (G-A-*O*-g); p-Coumaric acid *O*-glucoside (CA-*O*-g); Ferulic acid *O*-glucoside (FA-*O*-g); Quercetin 3-sambubioside-3′-glucoside (Q-S-g); Quercetin 3-glucosyl-(1->2)-galactoside (Q-G-g); Quercetin 3-(2G-xylosylrutinoside) (Q-G-X-r); Quercetin 3-*O*-rhamnoside 7-*O*-glucoside (Q-r-g). The pattern and magnitude relating to the colour intensity (hue) from +2 to −2 and 0 as symmetry) relating to visualization of response intensities of 19 compounds (identified and unidentified compounds) present in theses cowpea cultivars.

The unsupervised PCA illustrates clustering different cowpea cultivars (Figure S3), VOP1 cluster, the bigger cluster (VOP2-7) and VOP8 cluster. The PC 1 and PC 2, described for more than 70% of the variance, separated the cowpea cultivar VOP1 and VOP8 from the rest of the cultivars along the PC1 and PC2, respectively. A supervised orthogonal projection to latent structure-discriminant analysis (OPLS) model was performed in order to understand the separation of the clustered groups of cowpea cultivars, clearly based on their phenolic metabolites. In the *S*-plot (Figure 3), the compounds further along the *x*-axis contributed substantially to the variance between the groups, whilst the further the *Y*-axis, the higher the accuracy of the analytical result [15]. Therefore, in the *S*-plot, an unidentified compound ([M − H]^−^ 127.0020, *m*/*z* 2.82), presented in the upper right quadrant, showed the higher concentrations in cowpea cultivars VOP2, VOP3, VOP4, VOP5 VOP7 and VOP8 (Figure 4), whilst gentisicacid 5-*O*-glucoside, located at the lower left quadrant, showed higher concentration in cowpea cultivar VOP1. Thus, gentisicacid 5-*O*-glucoside is the marker candidate for the separation of VOP1 from the bigger cluster (VOP2-7) and the VOP8 which are not district from each other. Furthermore, the quantitative difference of the unidentified compound ([M − H]^−^ 127.0020) and gentisic acid 5-*O*-glucoside (eluted at Rt 2.82 and 3.27) revealed abundance at 50 and 200 peak intensity in counts/s respectively, in cowpea cultivar VOP1 (Figure S4). Samples from other cowpea cultivars, VOP2, VOP3, VOP4, VOP5, VOP7 and VOP8, demonstrated at Rt 2.82 and 3.27 the abundance of both unidentified compound ([M − H]^−^ 127.0020) and gentisic acid 5-*O*-glucoside at 50 peak intensity in counts/s (Figure S4).

### 3.3. In Vitro Antioxidant Capacity

**Figure 5:**
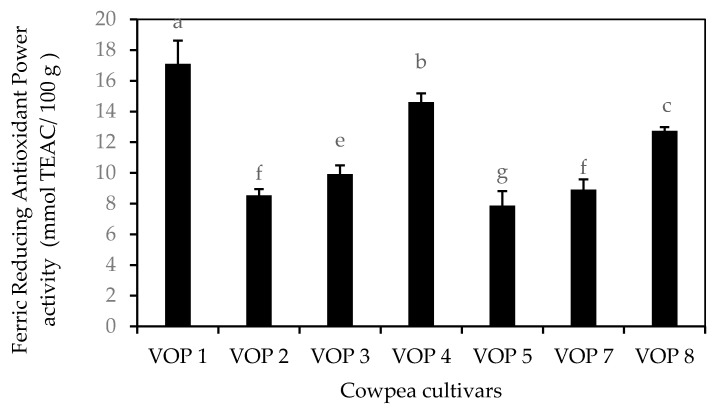
Antioxidant capacity of the leaves of different cowpea (leaves) cultivars. Bar with same alphabets are not significantly different between cultivars at *p* < 0.05. Data obtained were subjected to analysis of variance (ANOVA) using the statistical programme. Each bar represents the mean and standard deviation (*n* = 5).

FRAP assay was selected in this study because it is a quick and simple method to conduct, provides reproducible results and readily relates to the molar concentration of the antioxidants available in cowpea leaves. Results from in vitro antioxidant capacity (FRAP assay) are shown in Figure 5. Antioxidant capacity varied among the cultivars studied and VOP1 exhibited the strongest antioxidant capacity followed by VOP4. The FRAP activity in the leaf extracts of cowpea cultivar VOP1 is higher than that in indigenous fruits and vegetables, such as tree tomato (*Cyphomandra betacea*) at ripe stage (1.62 mmol TEC/100 g), and spider plant (*Cleome gynandra* L.; 1.56 mmol TEC/100 g) [28]. Indigenous vegetable amaranth leaves (*Amaranthus spinosus*; 1 mmol TEC/100 g) and commercial vegetable spinach, unknown cultivar (0.98 mmol TEC/100 g) [26], which showed lower FRAP activity than the leaves of cowpea cultivars VOP1, VOP4 and VOP8. Similarly, sweet potato leaves (*Solanum macrocarpon* L; 0.87 mmol TEC/100 g) [28] showed lower FRAP activity than the cowpea cultivars VOP1, VOP4, VOP8, VOP7 and VOP5.

Amongst the commercial fruits, banana (1.4 mmol TEC/100 g) and orange (1.2 mmol TEC/100 g) [28] demonstrated lower FRAP activity than the cowpea cultivars VOP1 and VOP4. Commercial fruit, papaya (0.89 mmol TEC/100 g) [28], showed a relatively similar level of FRAP activity as cowpea cultivars VOP2 and VOP7, but lower than VOP1, VOP4, VOP8, and VOP3. FRAP activity of passion fruit (7.2 mmol TEC/100 g) [28] similarly coincided with the activity of cowpea cultivar VOP5, however, all the leaves of other cowpea cultivars showed higher FRAP activity. Amongst the vegetables, brown beans (*Phaseolus vulgaris* L; 7.10 mmol TEC/100 g), sweet pepper (0.38 mmol TEC/100 g), tomato (0.38 mmol TEC/100 g), French beans (*Phaseolus vulgaris* L.; 0.21 mmol TEC/100 g), and sweet potato (*Ipomoea batatas* L. Lam.; 0.15 mmol TEC/100 g) [28] showed lower FRAP activity when compared to the leaves of all cowpea cultivars. In addition, cowpea cultivars VOP1 to VOP8 showed lower FRAP activity compared to seeds the seeds of Faba bean (*Vicia faba*) accessions that varied from 56.3 to 103.5 mmol TEC/100 g [29].

### 3.4. In Vitro Cytotoxic Effect

The cytotoxic effects of leaf extracts of cowpea cultivars on C2C12 myoblast cell line are given in percentage cell viability shown in Figure S5. All seven cowpea cultivars tested, using the C2C12 muscle cells, demonstrated absence of inhibitions on cell viability at 50% for the three concentrations 0.25, 0.5 and 1 mg/mL after 24 h incubation, whilst the highest toxicity was exhibited by the control (H_2_O_2_). Thus, all cowpea cultivars tested using this assay did not exhibit a strong enough toxicity to C2C12 myoblast cell lines at all tested concentrations.

### 3.5. Antidiabetic Effects and GLUT4 mRNA Levels

**Figure 6:**
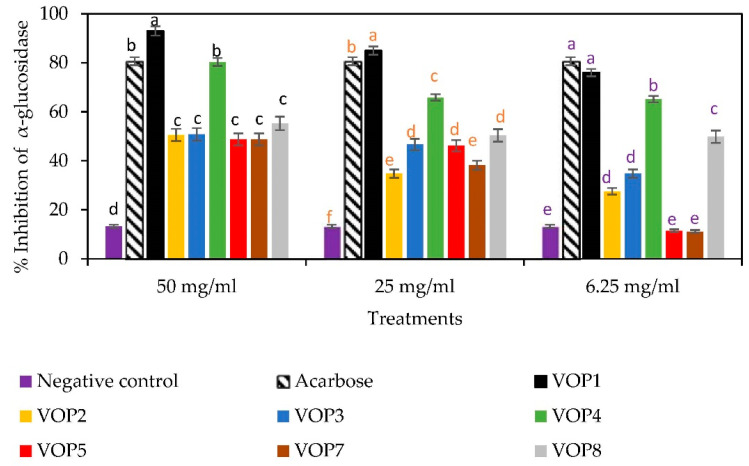
Percentage inhibition of leaf extracts of cowpea cultivars against α-glucosidase. Bars representing the enzyme activity for a specific leaf extract concentration with Bar with same alphabets are not significantly different between cultivars at *p* < 0.05. Data obtained were subjected to analysis of variance (ANOVA) using the statistical programme. Each bar represents the mean and standard deviation (*n* = 3).

Figure 6 illustrates the percentage inhibition of α-glucosidase of the leaf extracts of different cowpea cultivars at concentrations 6.25, 25 and 50 mg/mL using glucose as the substrate. The leaf extract of cowpea cultivar VOP1 at 25 and 50 mg/mL demonstrated the significantly higher percentage inhibitory values (86% and 93%) than the commercial inhibitor (Acarbose), whilst the leaf extract of VOP1 at 6.25 mg/mL revealed an almost similar percentage of inhibition (76%) as the commercial inhibitor (at 5 mg/mL). The cowpea leaf extracts of VOP4 at 50 mg/mL showed similar percentage of inhibition (80%) as the commercial inhibitor. The percentage inhibitory value of α-glucosidase in cowpea cultivar VOP4 was significantly lower than the VOP1 at all three concentrations tested; however, cowpea leaf extracts of the cultivar VOP4 showed higher inhibitory activity compared to cultivars VOP2, VOP3, VOP5, VOP7 and VOP8 at all the three tested levels.

**Figure 7:**
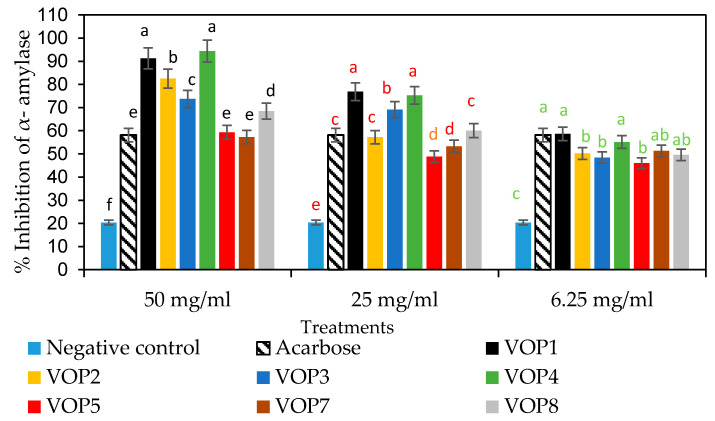
Percentage inhibition of leaf extracts of cowpea cultivars against α-amylase activities. Bars representing the enzyme activity for a specific leaf extract concentration. Bar with same alphabets are not significantly different between cultivars at *p* < 0.05. Data obtained were subjected to analysis of variance (ANOVA) using the statistical programme. Each bar represents the mean and standard deviation (*n* = 3).

The inhibition of α-amylase activity is shown in Figure 7. The leaf extracts of cowpea cultivars VOP1 and VOP4, at the concentration of 50 mg/mL, possessed the highest inhibitory activity (91–94%) compared to the commercial inhibitor (Acarbose), at the same level. A similar trend in results was also found for leaf extracts of cowpea accessions VOP1 and VOP4 at the intermediate concentration of 25 mg/mL, where they showed significantly higher inhibitory effects (75% and 76%) compared to the activity of the commercial inhibitor; however, leaf extracts of VOP1 and VOP4 at the lowest concentration of 6.25 mg/mL revealed a similar inhibitory effect (58%) as the commercial inhibitor. Overall, leaf extracts of cowpea cultivars VOP1 and VOP4 demonstrated the highest inhibitory effect on α-amylase and α-glucosidase activity among the cultivars studied.

Inhibition of enzymes, such as α-glucosidase and α-amylase, which are associated with carbohydrate digestion, is an important approach to reduce the postprandial hyperglycaemia [6]. Leaf extracts (at 50 mg/mL) of cowpea cultivar VOP1 revealed higher inhibitory activity of α-glucosidase and α-amylase compared to the leaf extracts of Moringa leaves (dried) [30]. Furthermore, cowpea cultivar VOP1 (50 µL/mL) showed more or less similar α-amylase inhibitory activity as blueberry cultivars, Blueray and Blur crop, grown in Southern Illinois, USA [31].

Rasouli et al. [32] explained that the presence of OH groups in positions 3 (ring C), 7 (ring A), 4 and 5 (ring B) in polyphenol molecular structure play a vital role in the inhibitory effects of the α-glucosidase and α-amylase activities. In addition, the total number of hydroxyl groups, C-2-C-3 double bond, and C-4 ketonic functional group play a major role in anti-diabetic effect. Furthermore, coumaric acid glycosides demonstrated greater inhibitory activities on these enzymes than the free (non-glyosidic) p-coumaric acid [33]. The observed difference in the degree of inhibition of these two enzymes could be due to the synergistic effect of different phenolic compounds and their varying concentrations [34].

**Figure 8:**
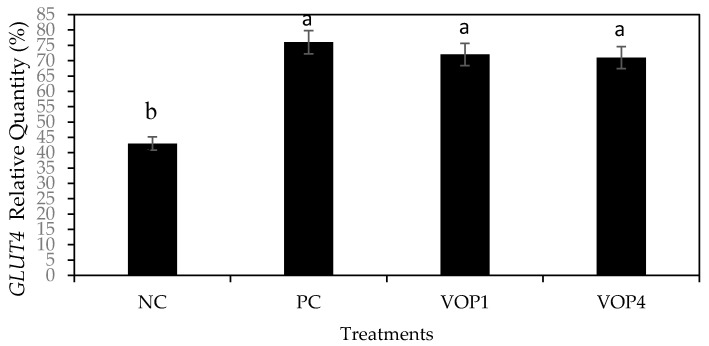
The *GLUT4* gene expression in C2C12 skeletal mouse muscle cells in presence of leaf extracts 50 mg/µL (100 µL) of cowpea cultivars VOP1 and VOP4 after 3 h of incubation. NC—negative control, PC—positive control (Insulin). Bar with same alphabets are not significantly different between cultivars at *p* < 0.05. Data obtained were subjected to analysis of variance (ANOVA) using the statistical programme. Each bar represents the mean and standard deviation (*n* = 3).

The influence of polyphenols of VOP1 and VOP4 on expression levels of *GLUT4* and *GAPDH* genes in C2C12 cells is shown in Figure 6. Leaf extracts of cowpea cultivars VOP4 significantly upregulated the *GLUT4* gene to a similar level as the comparative control treatment (insulin) (Figure 8). This result indicated higher glucose uptake by the C2C12 cells activated by the pool of phenolic compounds present in leaf extracts of cowpea cultivars VOP1 and VOP4. Boue et al. [35] demonstrated the influence of phenolic compounds on *GLUT4* mRNA levels in two pigmented rice bran extracts and stated the positive effects on long-term regulation of glucose transport.

Tea flavonol glycosides, which predominantly include quercetin 3-*O*-glucosyl-rhamnosyl-glucoside, showed significant differences with regard to glucose homeostasis in a type 2 diabetes mouse model after administration of flavonol-rich tea cultivars [36]. Ferulic acid, containing p-hydroxy and m-methoxy structures, was reported as one of the compounds that effectively enhanced insulin secretion [33]. This study indicates that the different phenolic compounds in cowpea leaves are responsible for the observed anti-diabetic activity, and this activity depends on the concentration of cowpea (VOP1 and VOP4) leaf extracts, and the specific molecular structure of the phenolic compounds.

### 3.6. Pearson’s Correlation Analysis

There were positive correlations between the antioxidant capacity (FRAP activity) and gentisic acid-5-*O*-glucoside, coumaric acid *O*-glucoside, ferulic acid *O*-glucoside, quercetin 3-glucosyl-(1->2)-galactoside, quercetin 3-sambubioside-3′-glucoside, Quercetin 3-(2G-xylosylrutinoside and quercetin 3-*O*-rhamnoside 7-*O*-glucoside (Table S2). Similarly, phenolic components, gentisic acid 5-*O*-glucoside, coumaric acid *O*-glucoside, ferulic acid *O*-glucoside, quercetin 3-glucosyl-(1->2)-galactoside, quercetin 3-sambubioside-3′-glucoside, quercetin 3-(2G-xylosylrutinoside) and quercetin 3-*O*-rhamnoside 7-*O*-glucoside from cowpea leaf extract revealed a positive correlation with α-glucosidase and α-amylase activity (Table S2). In addition, significantly positive correlations between FRAP activity and both α-glucosidase (R^2^ = 0.73, *p* < 0.05) and α-amylase (R^2^ = 0.80, *p* < 0.05) inhibition were also observed from the results of Pearson’s correlation analysis. It is evident from this study that the observed differences in antioxidant capacity between different cowpea cultivars could be related to the different concentrations of phenolic compounds. Although, antioxidant capacities and the concentrations of different phenolic compounds are affected by the geographical locations and altitude difference [37], in this case the plants were grown under the same environment and the observed differences in the concentrations of phenolic compounds and the antioxidant capacities are probably due to the genetic makeup of the cultivars.

### 3.7. Carotenoid Profile in Cowpea Cultivars

In general, cowpea cultivars VOP3 demonstrated significantly high total carotenoids content (Table 2), with lutein and β-carotene mainly contributing to the total carotenoid content. Table S3 illustrates the characterisation of carotenoid compounds detected in cowpea accessions by UHPLC–APCI-MS analysis. Figure S6A,B and Table S2 demonstrate the identification of the detected carotenoid components. Lutein concentration showed the following trend in cowpea cultivars: VOP2 > VOP8 > VOP3 > VOP1 > VOP4 > VOP7 > VOP5 (Table 3). Corn (0.092 mg/100 g), onion stalk (0.923 mg/100 g), broccoli (0.616 mg/100 g), capsicum (0.367 mg/100 g) [38], black Nightshade leaves (*Solanum nigrum*) (84.86 mg/100 g) [39] and carrot (42.0 mg/100 g) [40] had lower concentrations of lutein compared to the concentrations detected in cowpea cultivars. Zeaxanthin, was found at a minor proportion in cowpea cultivars, only ranged from 0.04 to 0.09 mg/100 g, which was lower than that reported in corn (0.28 mg/100 g) and onion stalk (0.305 mg/100 g) [41], but comparable to broccoli (0.04 mg/100 g) [41].Violaxanthin content was highest in VOP3 (24.9 mg/100 g), whilst the VOP5 showed the lowest violaxanthin content (8.4 mg/100 g) (Table 2). Onion stalks (1.83 mg/100 g), beetroot leaves (3.97 mg/100 g), carrot greens (7.00 mg/100 g) and broccoli (1.45 mg/100 g) [39] demonstrated lower violaxanthin content compared to the leaves of all cowpea cultivars. Conversely, coriander leaves (83.43 mg/100 g), amaranthus (*Amaranthus viridis*) (84.06 mg/100 g) [39], on dry weight basis, showed higher violaxanthin concentrations compared to the leaves of the studied cowpea cultivars. However, black Nightshade leaves (*Solanum nigrum*) (22.17 mg/100 g) [39] showed more or less similar concentrations of violoxanthin as VO3. Significantly, highest concentrations of all-trans-β-carotene were detected in cowpea cultivars VOP1, whereas VO5 showed the lowest concentrations (Table 2). Additionally, a trace amount of 9-*cis*-beta-carotene (2.4–3.6 mg/100 g) was also found in all cowpea cultivars (Table 2).

The results indicate that lutein and beta-carotene are predominant carotenoids in cowpea leaves, which are beneficial to human health. For instance, lutein and zeaxanthin are well known as important components of the human macula and retina [42]. Increased intake of lutein proved to correlate positively with increased macular pigment density, reducing the risk of macular degeneration by providing antioxidant protection against the damaging blue light [42]. The recommended daily levels for eye health are 10 mg/day of lutein and 2 mg/day of zeaxanthin for adults [43]. Thus, a serving of approximately 10 g of cowpea leaf powder added to a soup (except for VOP5) will fulfil the daily recommendation of lutein required for adults. However, it should be noted that the bioavailability of lutein depends on food preparation and cooking methods, therefore, follow up studies need to be investigated using both in vitro digestion models and human clinical trials to confirm the health-related benefits. Therefore, the cowpea cultivar VOP2 is promising for food supplementation programmes to reduce the risk of age-related macular degeneration.

Violaxanthin demonstrated higher antioxidant, anti-inflammatory and anti-proliferative activities [44]. All-*trans*-β-carotene is the predominant isomer in many fresh fruits and vegetables. The ratio of 9-*cis* to all-*trans-*β-carotene in papaya was higher (0.66) compared to the cowpea cultivars, VOP1 (0.034), VOP3 (0.042) and VOP8 (0.03). Furthermore, the *cis*-isomers were reported to increase during food processing, cooking methods and digestive metabolism in the intestine [45]. Previous reports based on different models suggest that the *cis*-isomers are preferred to *trans*-isomers and possess higher antioxidant potency [46].

### 3.8. Amino Acid Components in Cowpea Cultivars

The ratios of essential amino acids and branched amino acids to the total protein content were in the ranges of 36.7–40.8% and 17–19.8%, respectively (Table 3). The highest total protein content was detected in cowpea cultivars VOP3 (30.2 g/100 g) and VOP4 (31.3 g/100 g) compared to the other cultivars. Cowpea cultivars VOP1 showed the highest percentage of non-essential amino acids to the total protein content, whilst the percentage of essential amino acids to the total protein content was highest in cowpea cultivars VOP2, VOP3 and VOP7. Highest percentage of branched amino acids to the total protein content was detected in cowpea cultivars VOP2 and VOP7.

The results of amino acid analysis revealed that cowpea leaves contained both essential and non-essential amino acids and significant variation in the concentrations was the result of genotypic effects [47]. Non-essential amino acids, such as serine (*Ser*), arginine (*Arg*), glycine (*Gly*), aspartate (*Asp*), glutamate (*Glu*), alanine (*Ala*), proline (*Pro*), tyrosine (*Tyr*), were detected in all cowpea cultivars (Table 4). The *Asp* and *Glu* were identified as the predominant non-essential amino acids. Moreover, the cowpea cultivar VOP1 demonstrated the highest concentrations of *Asp*, followed by VOP4, VOP5 and VOP8. Cowpea cultivars VOP3, VOP4, VOP5 and VOP7 had the highest concentration of *Glu* compared to the other cowpea cultivars (Table 4).

Essential amino acids, histidine (*His*), threonine (*Thr*), lysine (*Lys*), methionine (*Met*), valine (*Val*), isoleucine (*lle*), leucine (*Leu*), phenylalanine (*phe*) were detected in all cowpea cultivars (Table 4), in which *Leu* was found as the predominant essential amino acid. Cowpea accessions VOP3, VOP4, VOP5 and VOP7 contained the highest concentration of *Leu*, whereas cowpea cultivars VOP3 and VOP4 contained the highest concentration of *Phe* (Table 4). Cowpea cultivar VOP7 was rich in *Lys,* followed by VOP4, VOP5 and VOP8. A moderately higher concentration of *Val* was detected in cowpea cultivars VOP3, VOP4, VOP5 and VOP7 (Table 4). Whilst *His* and *Met* were detected at lower concentrations in all cowpea cultivars, VOP4 and VOP3 contained the highest concentrations of *His* (Table 4). The trend or pattern in amino acid composition could relate to possible inherent differences between genotypes/cultivars. The more or less similar trends observed regarding the concentrations of essential amino acids in cowpea cultivars VOP3 and VOP4 probably confirm that these cultivars are genetically similar compared to the other cultivars. The amount of amino acids in cowpea leaves is lower than the cowpea grains [39], which supports the findings of the present study.

The daily requirement of essential amino acid intake from cowpea cultivars VOP3 or VOP4 was calculated based on the FAO/WHO/UNU [46] guidelines, suggesting that a 110.95 g serving portion of cowpea accessions VOP3 or VOP4 is able to fulfil the daily requirement of *Phe*, *Leu* and *lle* for adults with 70 kg body weight. Similarly, 20.95 g of serving portion of leaves of the corresponding cowpea cultivars fulfils the daily requirement of *Thr* for adults (70 kg body weight). Thus, cowpea cultivars VOP3 and VOP4 showed potential to fulfil the daily requirements of some essential amino acids.

## 4. Conclusions

This study illustrated the carotenoid and amino acid profile in different cowpea accessions grown in Africa. Leaves of cowpea cultivar VOP2 are a rich source of lutein. Concentration of identified phenolic compounds varied among the cowpea cultivars. Chemomertic analysis indicated, based on the phenolic metabolites, that cowpea accession VOP1 significantly differed from the rest. The Pearson correlation test results showed that gentisic acid-5-*O*-glucoside, quercetin 3-(2G-xylosylrutinoside) and quercetin 3-glucosyl-(1->2)-galactoside in cowpea cultivars VOP1 and VOP4 might be most responsible for the observed in vitro α- amylase and α-glucosidase activities. Leaf extracts of cowpea cultivars VOP1 and VOP4 enhanced the upregulation of glucose transporter *GLUT4* gene and showed similar anti-hyperglycaemic effects to insulin. This study further confirms the relationship between cowpea leaf phytochemicals and glucose metabolism/diabetes.

## Figures and Tables

**Table 1:** Identification of phenolic compounds in different cowpea cultivars by a UPLC–QTOF/MS data.

Peak	Retention Time	M-H	M-H Formula	ppm Error	MSE Fragments	UV	Identification
1	3.29	315.0697	C_13_H_15_O_9_	−5.1	152,108	152,108	Gentisic acid 5-*O*-glucoside
2	4.51	325.0889	C_15_H_17_O_8_	−1.2	163,145,119	289	Coumaric acid *O*-glucoside
3	4.84	355.1024	C_16_H_19_O_9_	−1.4	261,243,193,175,160,134	321	Ferulic acid *O*-glucoside
4	5.33	757.1812	C_57_H_25_O_3_	1.1	301,271,197	255,351	Quercetin 3-sambubioside-3′-glucoside
5	5.60	625.1356	C_34_H_25_O_12_	1.6	301,284,271,255,178,155	255	Quercetin 3-glucosyl-(1->2)-galactoside
6	5.70	741.1879	C_32_H_37_O_20_	0.1	625,443,355,285	265,335	Quercetin 3-(2G-xylosylrutinoside)
7	5.95	609.1493	C_27_H_29_O_16_	3.8	595,361,301,271,255	257,333	Quercetin 3-*O*-rhamnoside 7-*O*-glucoside

**Table 2:** Carotenoid content in the leaves of different cowpea cultivars.

Cowpea Cultivars	Violaxanthin	Lutein	Zeaxanthin	All-Trans-Beta-Carotene	9-Cis-Beta-Carotene	Total Carotenoids
	mg/100 g DW					
VPO1	17.8 ± 1.5 *^,c^	109.1 ± 8.6^d^	0.04 ± 0.01^d^	92.6 ± 1.7^a^	3.2 ± 0.2^b^	222.7 ± 1.0^b^
VPO2	20.4 ± 0.7^b^	124.6 ± 1.8^a^	0.06 ± 0.02^c^	71.1 ± 1.7^d^	3.0 ± 2.6^c^	220.7 ± 1.6^c^
VPO3	24.9 ± 6.0^a^	111.2 ± 1.7^c^	0.04 ± 0.01^d^	84.7 ± 5.9^c^	3.6 ± 0.3^a^	224.5 ± 1.0^a^
VPO4	16.9 ± 0.7^d^	99.9 ± 9.5^e^	0.06 ± 0.01^c^	59.3 ± 8.7^e^	3.3 ± 0.2^b^	179.6 ± 1.5^e^
VPO5	8.4 ± 1.3^f^	74.5 ± 1.0^g^	0.10 ± 0.03^a^	43.0 ± 4.9^f^	2.4 ± 0.1^e^	129.7 ± 1.5^f^
VPO7	15.2 ± 1.9^e^	94.3 ± 1.0^f^	0.04 ± 0.04^d^	70.3 ± 1.3^d^	2.7 ± 0.1^d^	181.8 ± 1.0^d^
VPO8	15.5 ± 1.1^e^	116.5 ± 1.2^b^	0.09 ± 0.01^b^	87.2 ± 5.2^b^	2.9 ± 0.1^d^	222.2 ± 1.5^b^

DW—dry weight, * Data present mean and standard deviation. Different letters at the same column indicate significant differences at (*p* < 0.05).

**Table 3:** Percentage of total protein, non-essential, essential and branched amino acids in different cowpea cultivars.

Cowpea Cultivars	Total Protein (g/100 g DW)	% Non-Essential Amino Acids	% Essential Amino Acids	% Branched-Chain Amino Acids
VOP1	28.4 ± 0.1 *^,d^	63.61 ± 0.2^a^	36.7 ± 0.2^c^	17.0 ± 0.2^d^
VOP2	25.1 ± 0.1^f^	59.20 ± 0.1^d^	40.8 ± 0.1^a^	19.8 ± 0.0^a^
VOP3	30.2 ± 0.2^b^	59.40 ± 0.1^d^	40.6 ± 0.1^a^	18.4 ± 0.3^bc^
VOP4	31.3 ± 0.3^a^	61.30 ± 0.7^c^	38.7 ± 0.7^b^	18.1 ± 0.6^c^
VOP5	29.3 ± 0.2^c^	62.50 ± 0.3^b^	37.5 ± 0.3^c^	18.6 ± 0.3^bc^
VOP7	28.3 ± 0.2^d^	59.50 ± 0.2^d^	40.5 ± 0.2^a^	19.2 ± 0.1^ab^
VOP8	27.2 ± 0.3^e^	61.50 ± 0.3^bc^	38.5 ± 0.3^b^	17.9 ± 0.2^b^

* Data present mean and standard deviation (*n* = 3). Different letters at the same column indicate significant differences at (*p* < 0.05).

**Table 4:** Non-essential and essential amino acids in cowpea cultivars.

	**Non-Essential Amino Acids (g/100 g DW)**							
Cowpea cultivars	*Ser*	*Arg*	*Gly*	*Asp*	*Glu*	*Ala*	*Pro*	*Try*
VOP1	1.38 ± 0.41b	1.92 ± 0.20c	1.33 ± 0.26c	5.96 ± 0.31a	3.28 ± 0.12bc	1.44 ± 0.34c	1.32 ± 0.25b	1.30 ± 0.14b
VOP2	1.29 ± 0.23b	1.96 ± 0.12c	1.28 ± 0.17c	3.46 ± 0.10c	3.07 ± 0.38c	1.42 ± 0.21c	1.13 ± 0.34c	1.20 ± 0.21b
VOP3	1.60 ± 0.30a	2.42 ± 0.25a	1.71 ± 0.11a	3.49 ± 0.26c	3.67 ± 0.40a	1.56 ± 0.16b	1.43 ± 0.31a	1.91 ± 0.30a
VOP4	1.70 ± 0.34a	2.52 ± 0.20a	1.62 ± 0.20a	4.80 ± 0.21b	3.47 ± 0.51b	1.58 ± 0.24b	1.45 ± 0.11a	2.01 ± 0.42a
VOP5	1.42 ± 0.20b	2.20 ± 0.31b	1.53 ± 0.15b	4.96 ± 0.18b	3.88 ± 0.12a	1.64 ± 0.10ab	1.46 ± 0.20a	1.24 ± 0.20b
VOP7	1.40 ± 0.42b	1.82 ± 0.40c	1.50 ± 0.31b	3.92 ± 0.11c	3.73 ± 0.11a	1.74 ± 0.50a	1.44 ± 0.23a	1.27 ± 0.10b
VOP8	1.37 ± 0.21b	1.80 ± 0.30c	1.36 ± 0.20bc	4.63 ± 0.09b	3.42 ± 0.27b	1.53 ± 0.34bc	1.25 ± 0.12b	1.36 ± 0.20b
	**Essential amino acids (g/100 g DW)**							
	*His*	*Thr*	*Lys*	*Met*	*Val*	*lle*	*Leu*	*phe*
VOP1	0.50 ± 0.16b	1.41 ± 0.27bc	1.75 ± 0.20c	0.31 ± 0.70 ^**^	1.26 ± 0.51c	1.41 ± 0.32c	2.13 ± 0.32cd	1.60 ± 0.10b
VOP2	0.51 ± 0.29b	1.31 ± 0.10c	1.71 ± 0.17c	0.30 ± 0.50	1.28 ± 0.41c	1.22 ± 0.17d	2.05 ± 0.17d	1.41 ± 0.20c
VOP3	0.72 ± 0.20a	1.67 ± 0.30a	1.51 ± 0.41d	0.32 ± 0.14	1.56 ± 0.12a	1.54 ± 0.12b	2.46 ± 0.15a	2.45 ± 0.23a
VOP4	0.66 ± 0.18a	1.65 ± 0.25a	1.39 ± 0.32e	0.25 ± 0.28	1.55 ± 0.30a	1.66 ± 0.25a	2.44 ± 0.31ab	2.46 ± 0.16a
VOP5	0.45 ± 0.10b	1.39 ± 0.60bc	1.91 ± 0.50b	0.29 ± 0.12	1.54 ± 0.18a	1.58 ± 0.50ab	2.35 ± 0.40b	1.48 ± 0.17c
VOP7	0.46 ± 0.38b	1.49 ± 0.10b	2.04 ± 0.27a	0.38 ± 0.32	1.53 ± 0.10a	1.41 ± 0.12c	2.47 ± 0.51a	1.65 ± 0.32b
VOP8	0.51 ± 0.41b	1.40 ± 0.31bc	1.92 ± 0.15b	0.27 ± 0.41	1.39 ± 0.21b	1.30 ± 0.10d	2.19 ± 0.30c	1.49 ± 0.12b

DW—dry weight. Data present mean and standard deviation (*n* = 3). Different letters at the same column indicate significant differences at (*p* < 0.05). ** are not significant.

## Supplementary Materials

The following are available online at https://www.mdpi.com/2304-8158/9/9/1285/s1, Figure S1: One representative UPLC–Q-TOF/MS chromatogram illustrating the predominant phenolic compounds in the leaves of cowpea cultivars VOP1, VOP2, VOP3, VOP4, VOP5, VOP7 and VOP8, Figure S2: MS spectra, Figure S3: Score plot of Principal component analysis (unsupervised) based on UPLC–Q-TOF/MS spectra of the leaves of different cowpea cultivars, Figure S4: Histogram illustrating quantitative differentiation of biomarkers between the Cowpea cultivar VOP1 and other cowpea cultivarsVOP2, VOP3, VOP4, VOP5, VOP7, and VOP8, Figure S5: Percentage cell viability of C2C12 myoblast cell lines exposed to three different concentrations of leaf extracts of cowpea cultivars, Figure 6A: Representative UV chromatogram of carotenoids in cowpea leaves at @450 nm, Figure 6B: Representative TIC and mass features of individual carotenoid compounds detected in cowpea leaves, Table S1. Primer sequences used to amplify, the GLUT4 and GAPDH cDNA, Table S2: Pearson’s correlation coefficients between targeted phenolic components and in vitro antioxidant (FRAP), α-glucosidase and α-amylase activities, Table S3 Characterization of carotenoid compounds detected in cowpea accessions by LC–APCI-MS scanning at positive mode.
