# Supplementary material for: Comparison of Phenolic Compounds, Carotenoids, Amino Acid Composition, In Vitro Antioxidant and Anti-Diabetic Activities in the Leaves of Seven Cowpea (Vigna unguiculata) Cultivars
Source: Foods. 2020 Sep 12;9(9):1285. doi: 10.3390/foods9091285 (PMC7554895; doi:10.3390/foods9091285)
Supplement: Supplementary file 1 [file foods-09-01285-s001.pdf]

Comparison of phenolic compounds, carotenoids, amino acid composition, *in vitro* antioxidant and anti-diabetic activities in the leaves of seven cowpea (*Vigna unguiculata*) cultivars

Mapula R. Moloto,[1] Anh Dao T. Phan,[2] Jerry L. Shai,[3] Yasmina Sultanbawa,[2] Dharini Sivakumar[1,2] *

[1] Phytochemical Food Network Research Group, Department of Crop Sciences, Tshwane University of Technology, Pretoria West, 0001, South Africa; mapularebahlotsem@gmail.com (MM);SivakumarD@tut.ac.za (DS)

[2] ARC Industrial Transformation Training Centre for Uniquely Australian Foods, Queensland Alliance for Agriculture and Food Innovation, The University of Queensland, QLD 4108, Queensland, Australia; y.sultanbawa@uq.edu.au [YS]; a.phan1@uq.edu.au [AP]

[3] Department of Biomedical Sciences, Tshwane University of Technology, Arcadia, Pretoria 0001, South Africa; ShaiLJ@tut.ac.za [JS]

*Corresponding author DS SivakumarD@tut.ac.za

Supplementary files

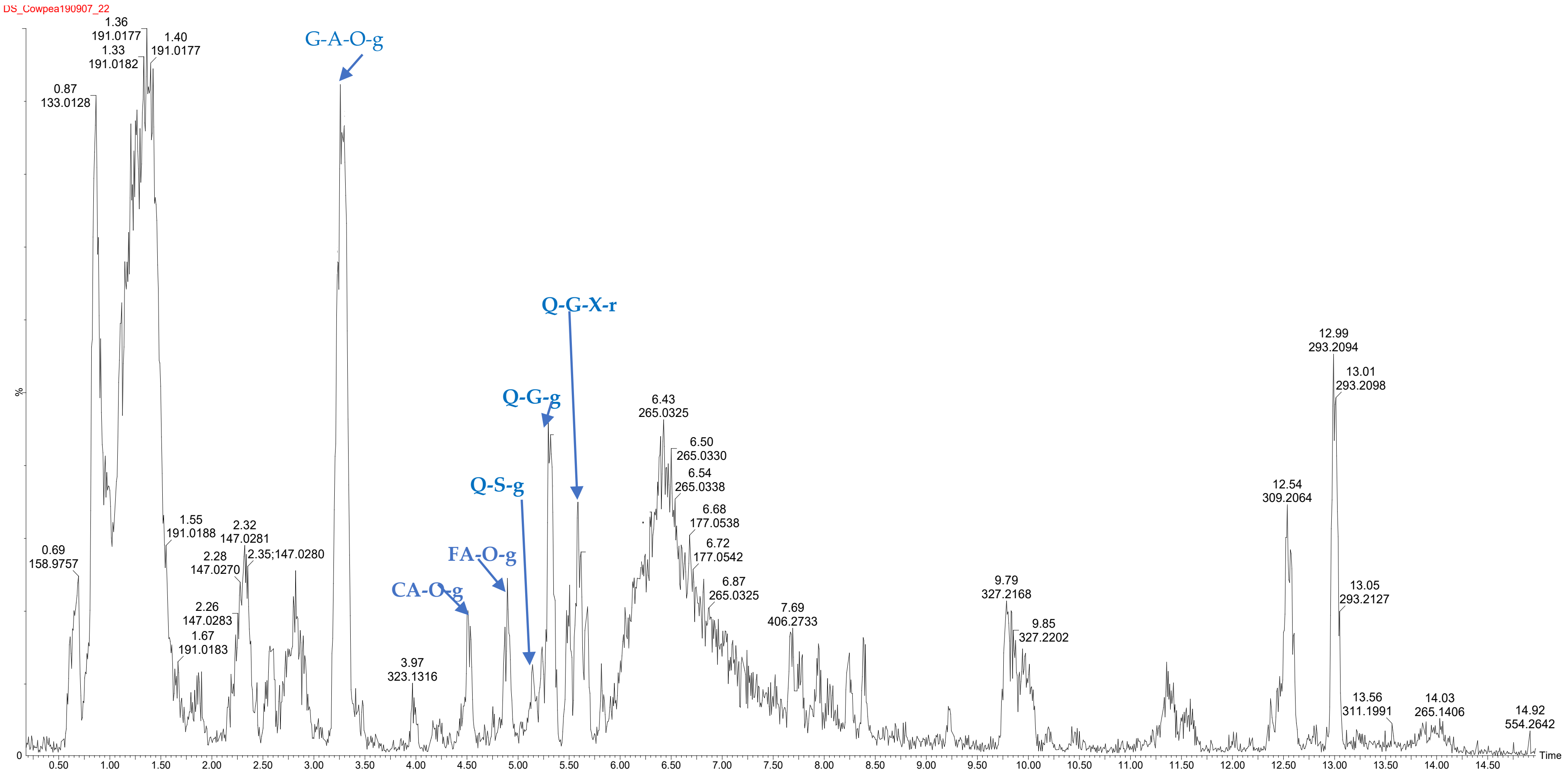

Supplementary Figure 1. One representative UPLC–Q-TOF/MS chromatogram illustrating the predominant phenolic compounds in the leaves of cowpea cultivars VOP1, VOP2, VOP3, VOP4, VOP5, VOP7 and VOP8. The chromatograms were constructed using three replicates of cumulative samples. The relative peak intensity is normalized, and peaks are expressed as the percentage highest peak intensity. Gentisic acid 5-O-glucoside (G-A-O-g); p-Coumaric acid O-glucoside (CA-O-g); Ferulic acid O-glucoside (FA-O-g); Quercetin 3-sambubioside-3'-glucoside (Q-S-g); Quercetin 3-glucosyl-(1->2)-galactoside (Q-G-g); Quercetin 3-(2G-xylosylrutinoside) (Q-G-X-r); Quercetin 3-O-rhamnoside 7-O-glucoside (Q-r-g)

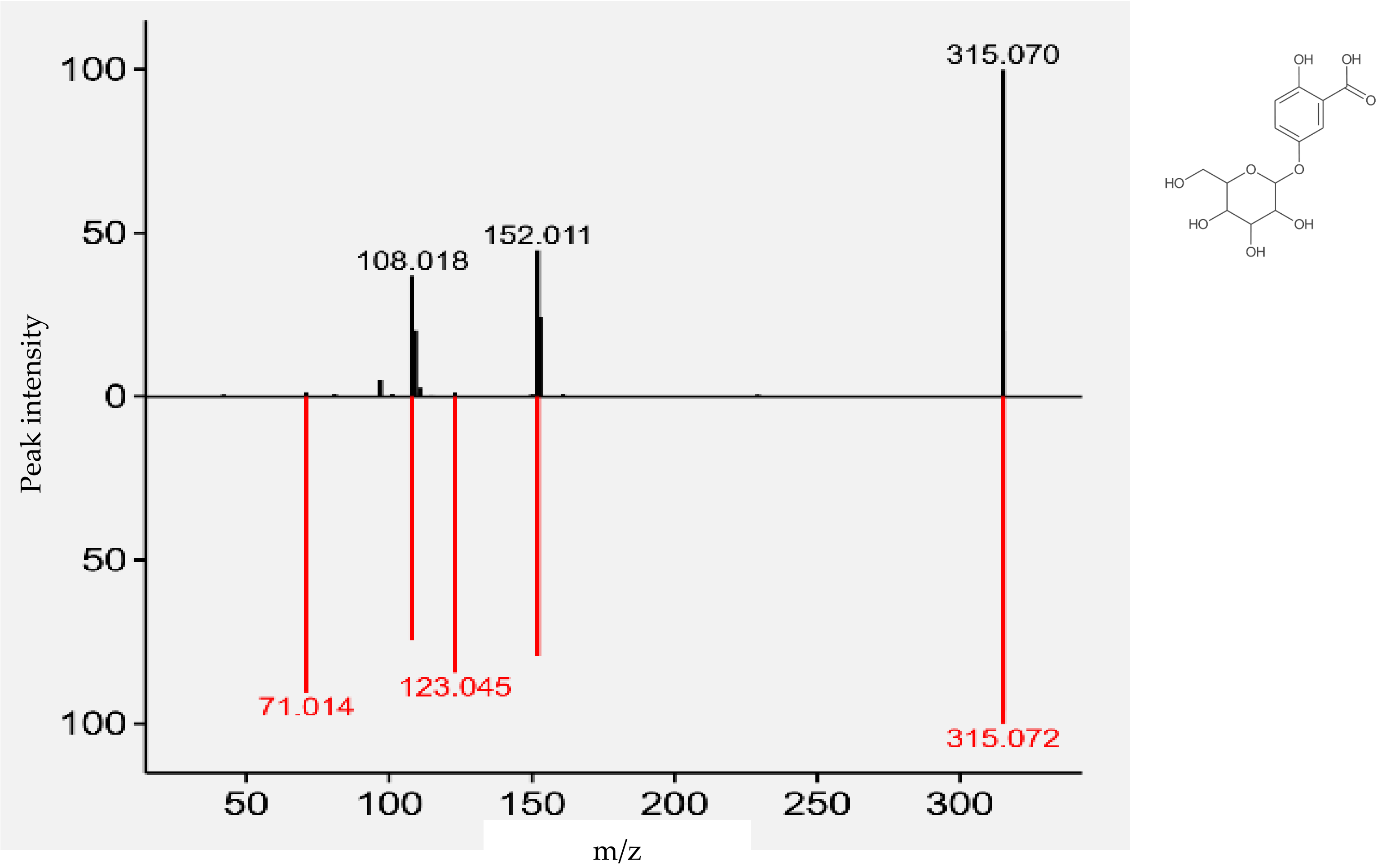

Supplementary Figure 2A. MS spectra of gentisic acid-5-O-glucoside

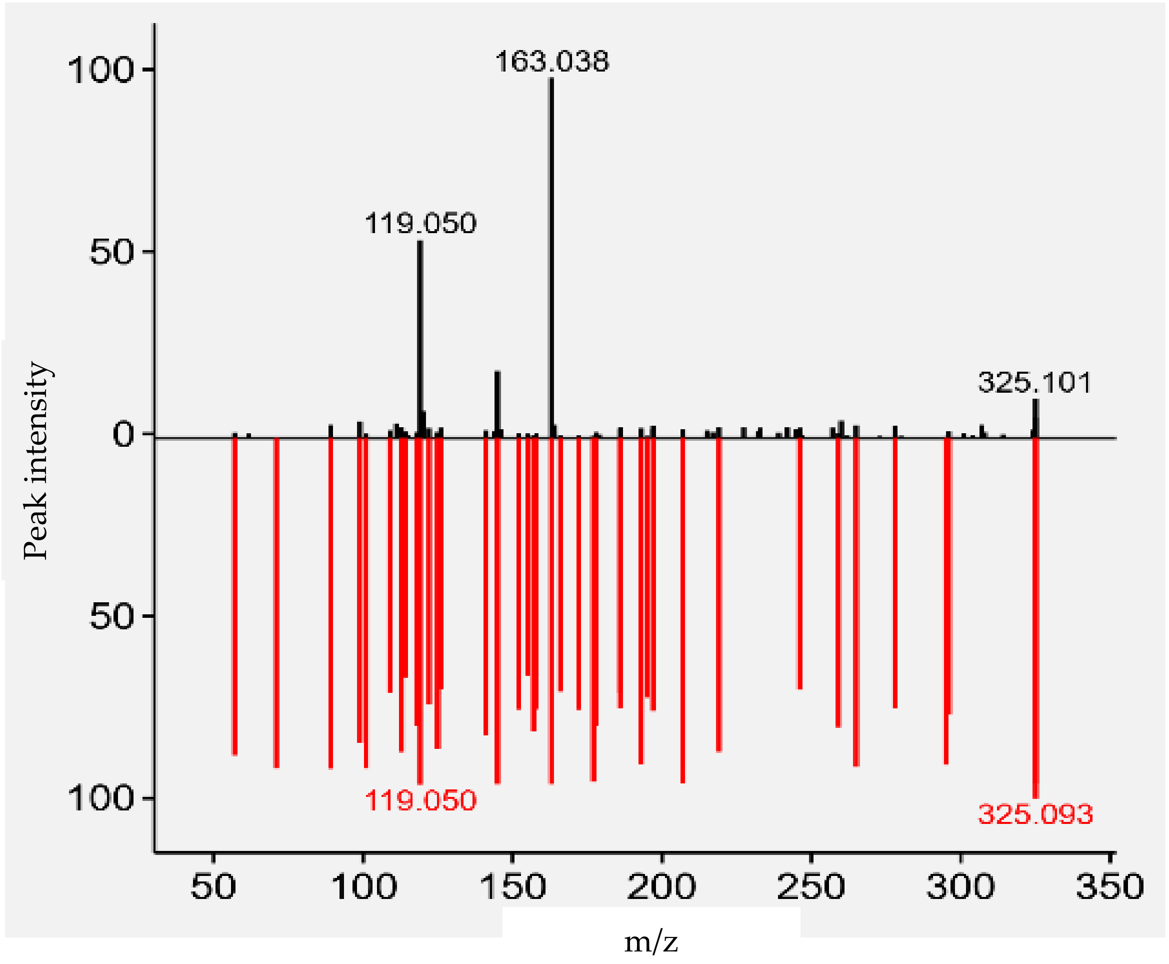

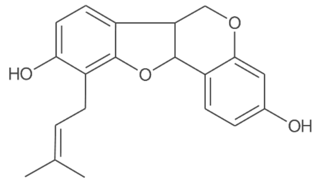

Supplementary Figure 2B. MS spectra of coumaric acid-O-glucoside

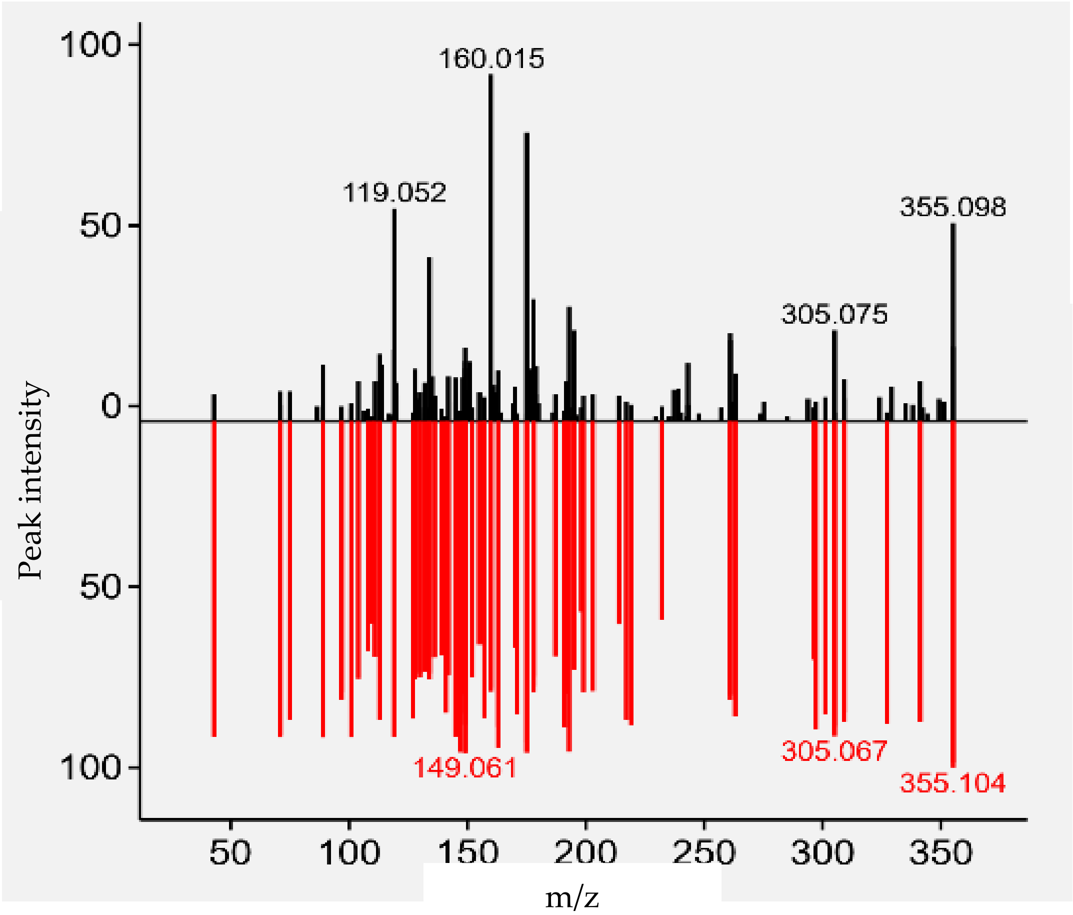

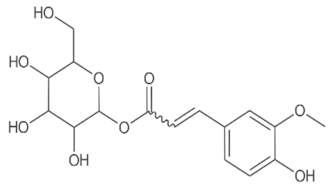

Supplementary Figure 2C. MS spectra of ferulic acid-O-glucoside

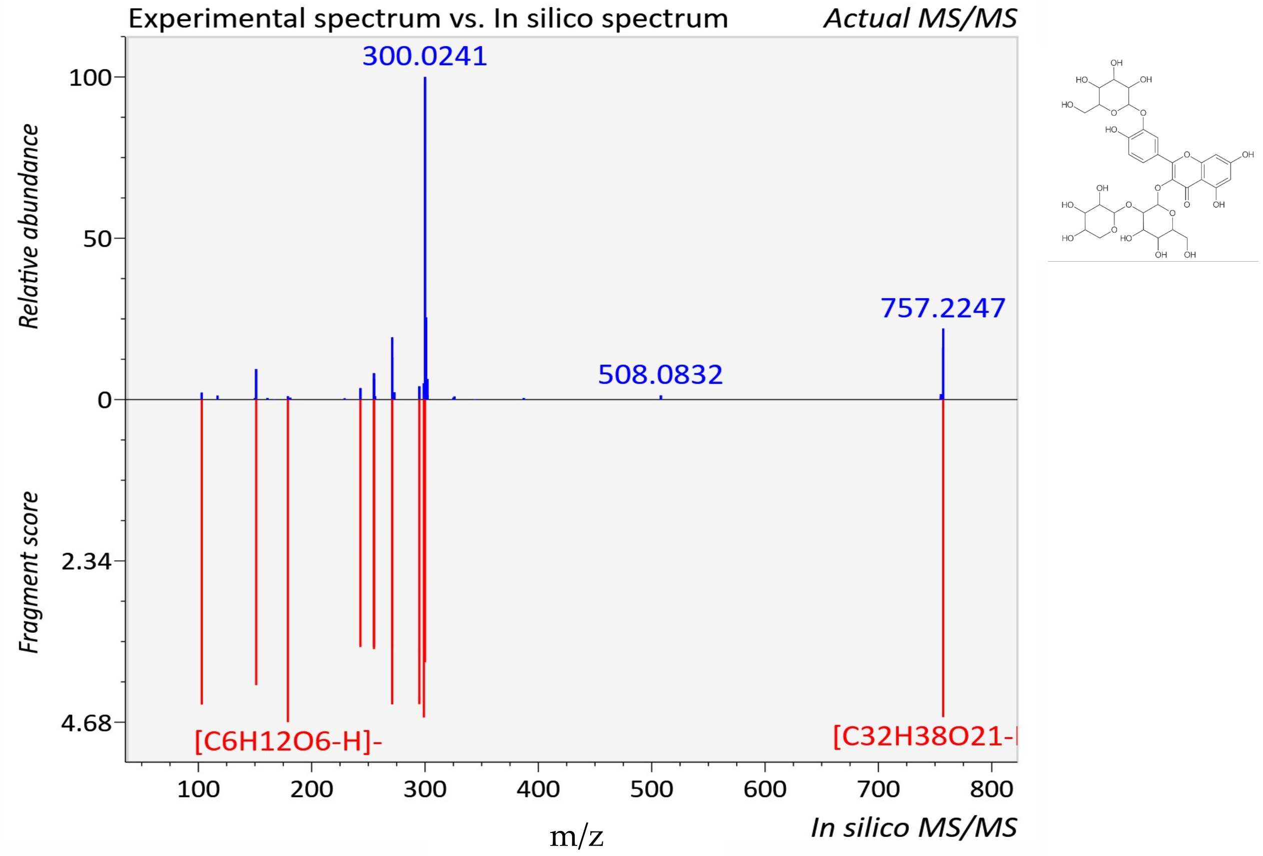

Supplementary Figure 2D. MS spectra of quercetin 3-sambubioside-3'-glucoside

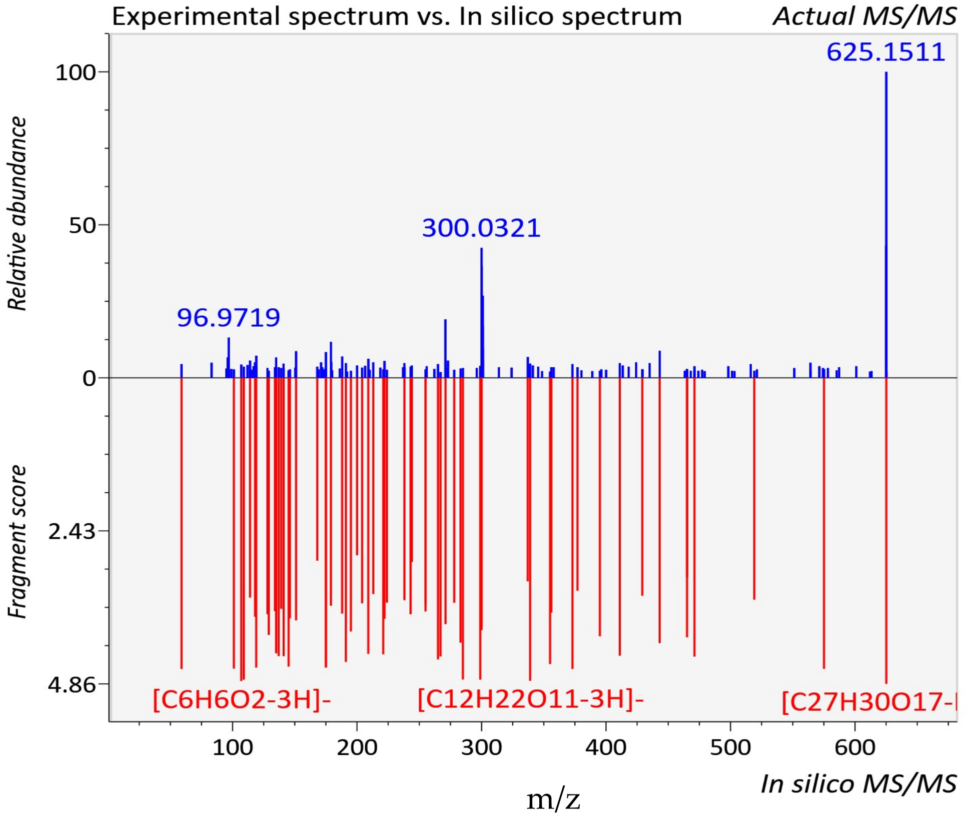

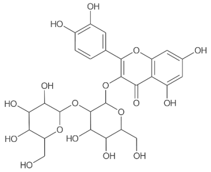

Supplementary Figure 2E. MS spectra of quercetin 3-glucosyl-(1->2)-galactoside

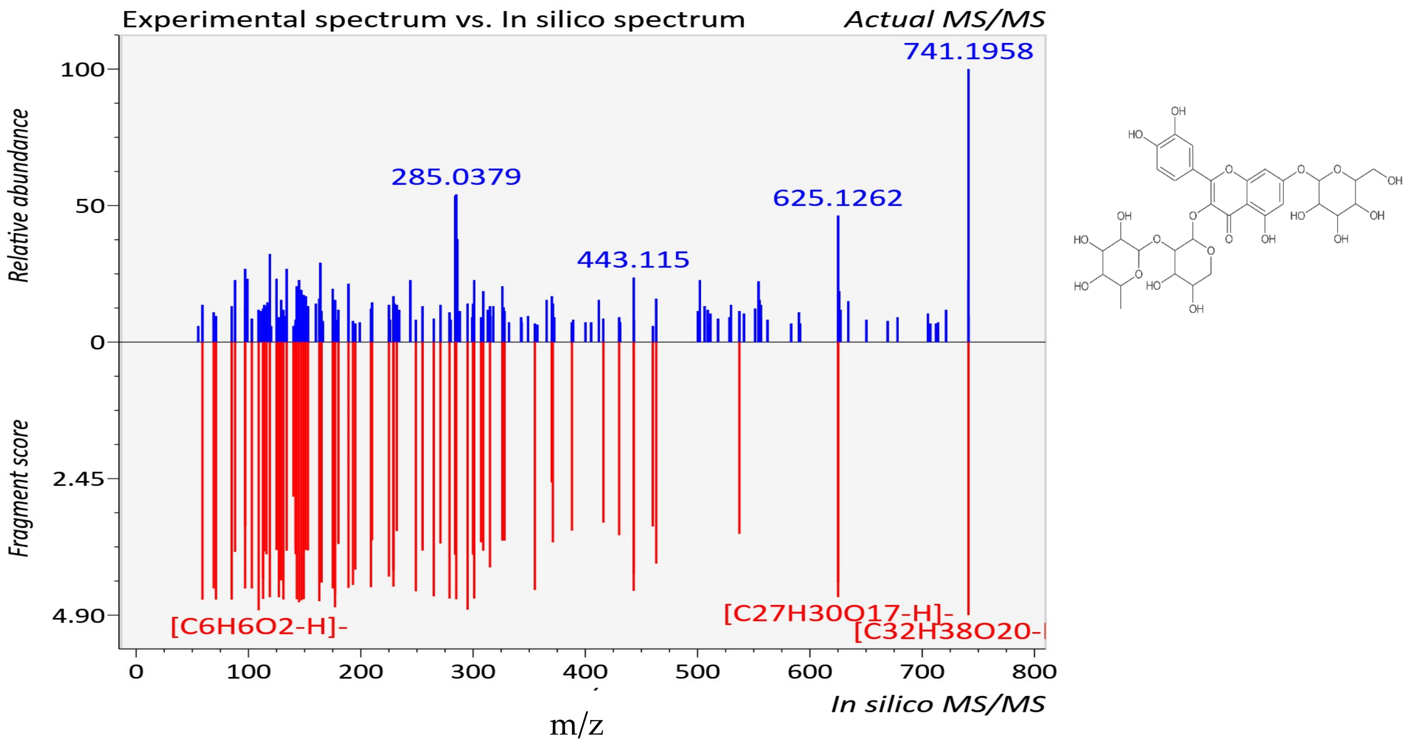

Supplementary Figure 2F. MS spectra of Quercetin 3-(2G-xylosylrutinoside)

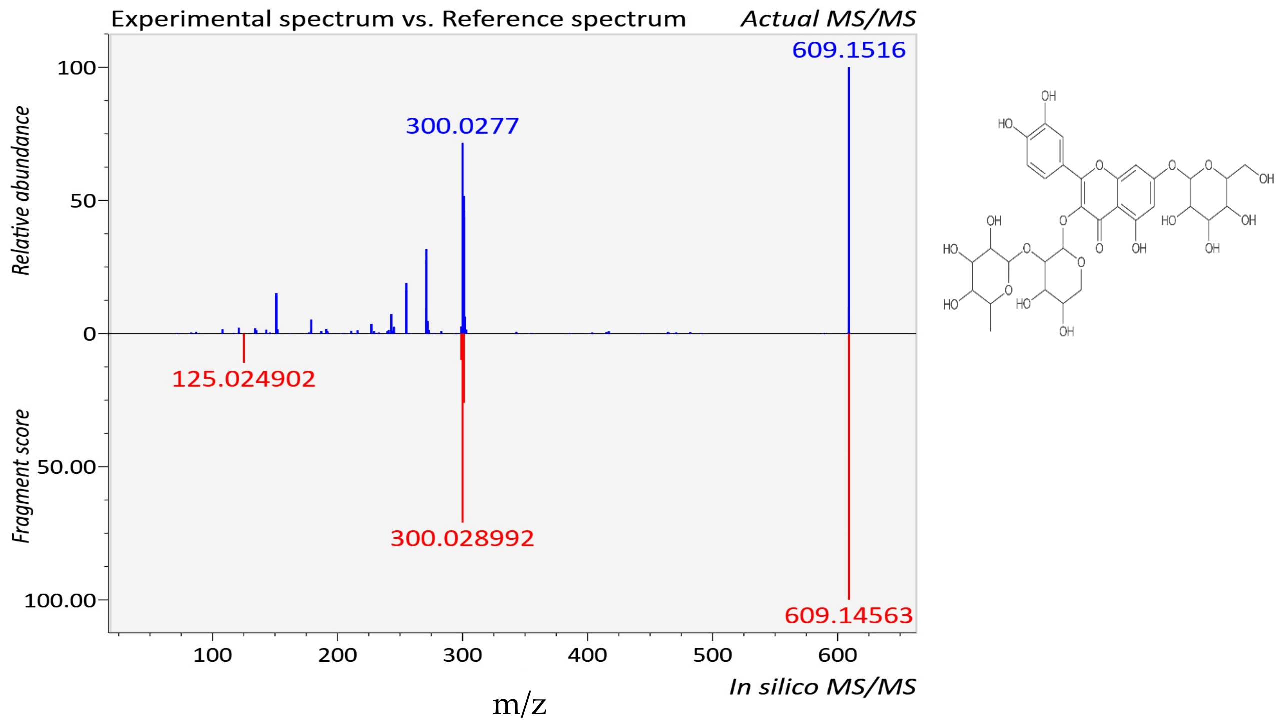

Supplementary Figure 2G. MS spectra of quercetin 3-O-rhamnoside 7-O-glucoside

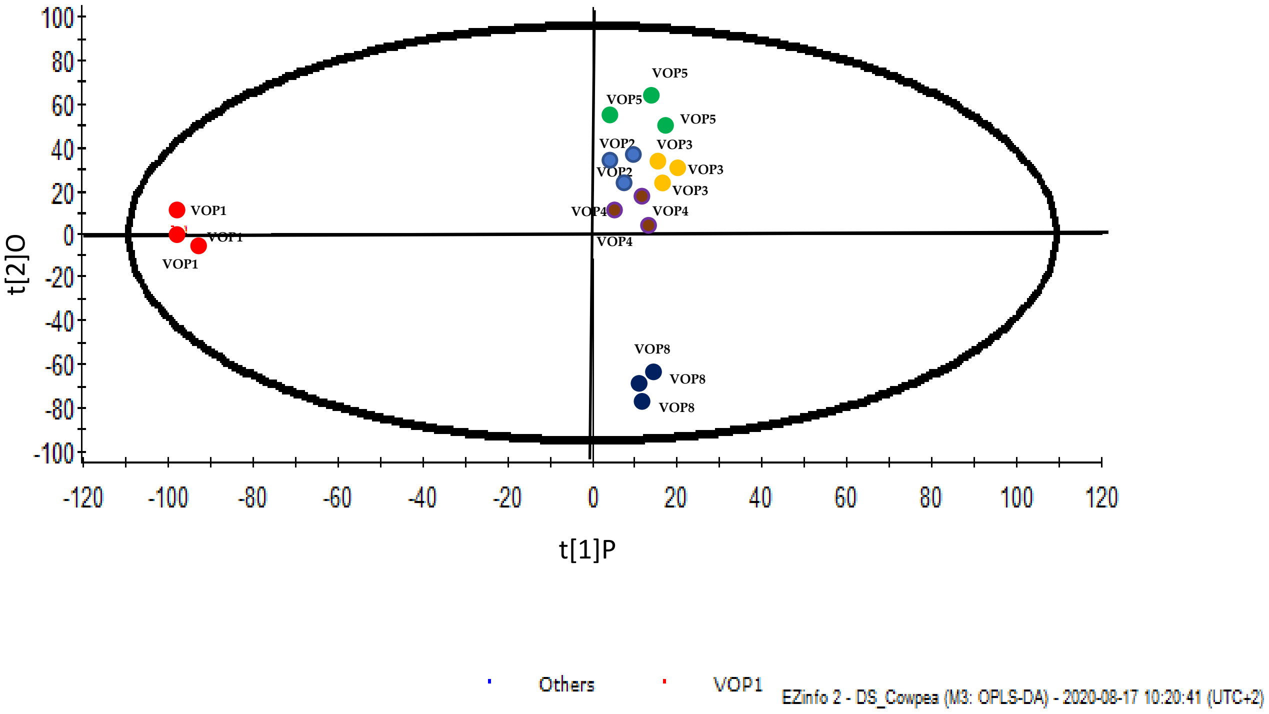

Supplementary Figure. 3 Score plot of Principal component analysis (unsupervised) based on UPLC–Q-TOF/MS spectra of the leaves of different cowpea cultivars

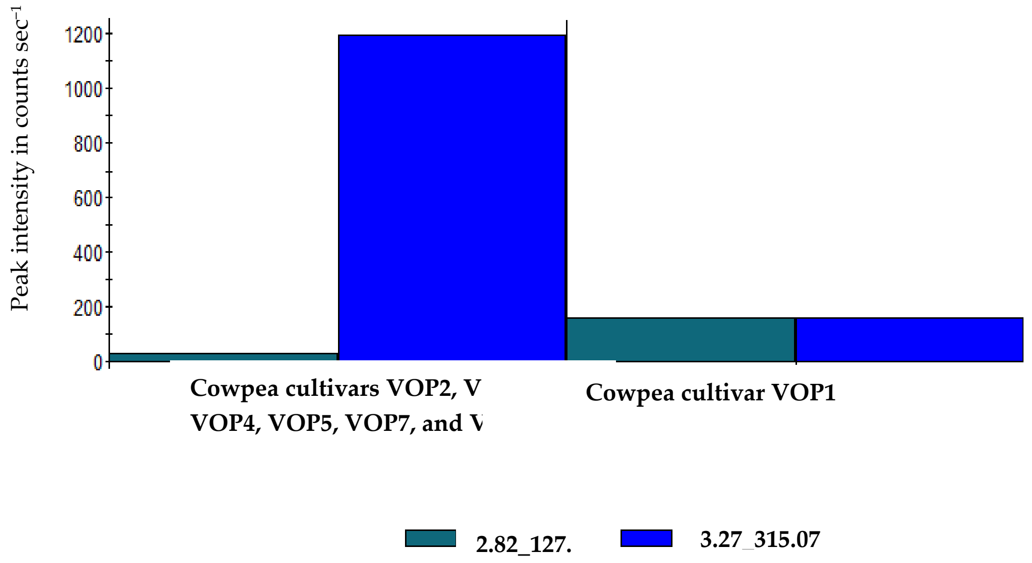

Supplementary Figure 4. Histogram illustrating quantitative differentiation of biomarkers between the Cowpea cultivar VOP1 and other cowpea cultivarsVOP2, VOP3, VOP4, VOP5, VOP7, and VOP8

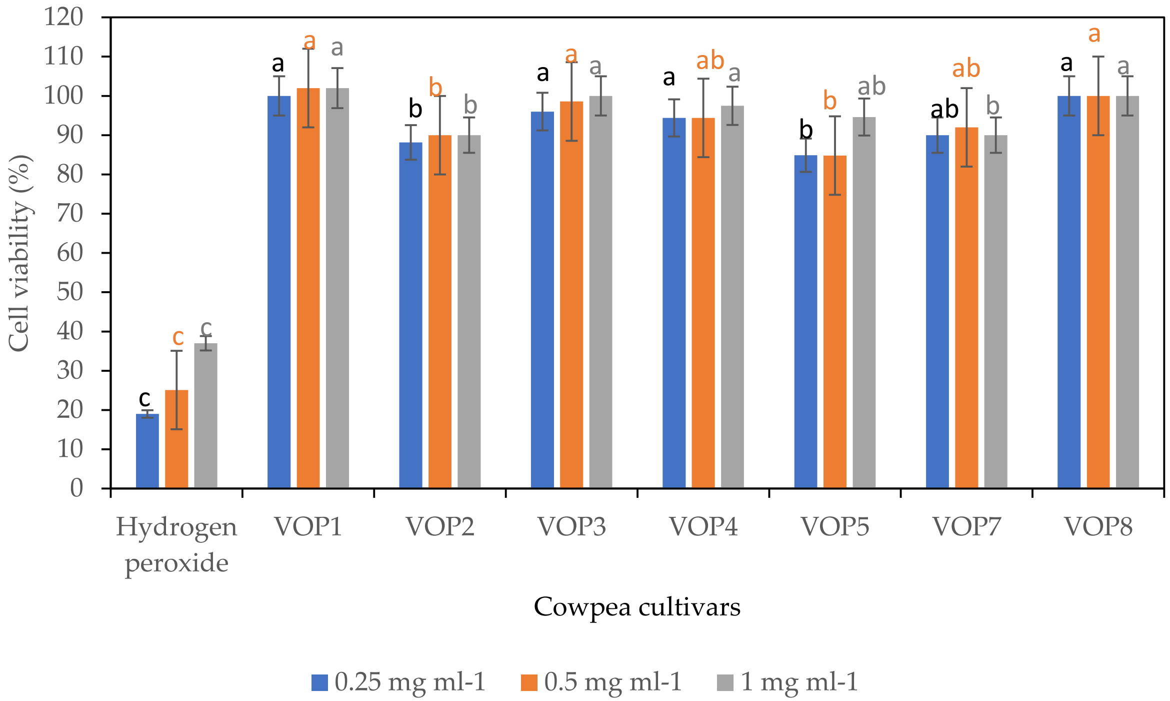

Supplementary Figure 5 Percentage cell viability of C2C12 myoblast cell lines exposed to three different concentrations of leaf extracts of cowpea cultivars

Bar with same alphabets are not significantly different between cultivars at $p < 0.05$ for a specific phenolic compound. Data obtained were subjected to analysis of variance (ANOVA) using the statistical programme

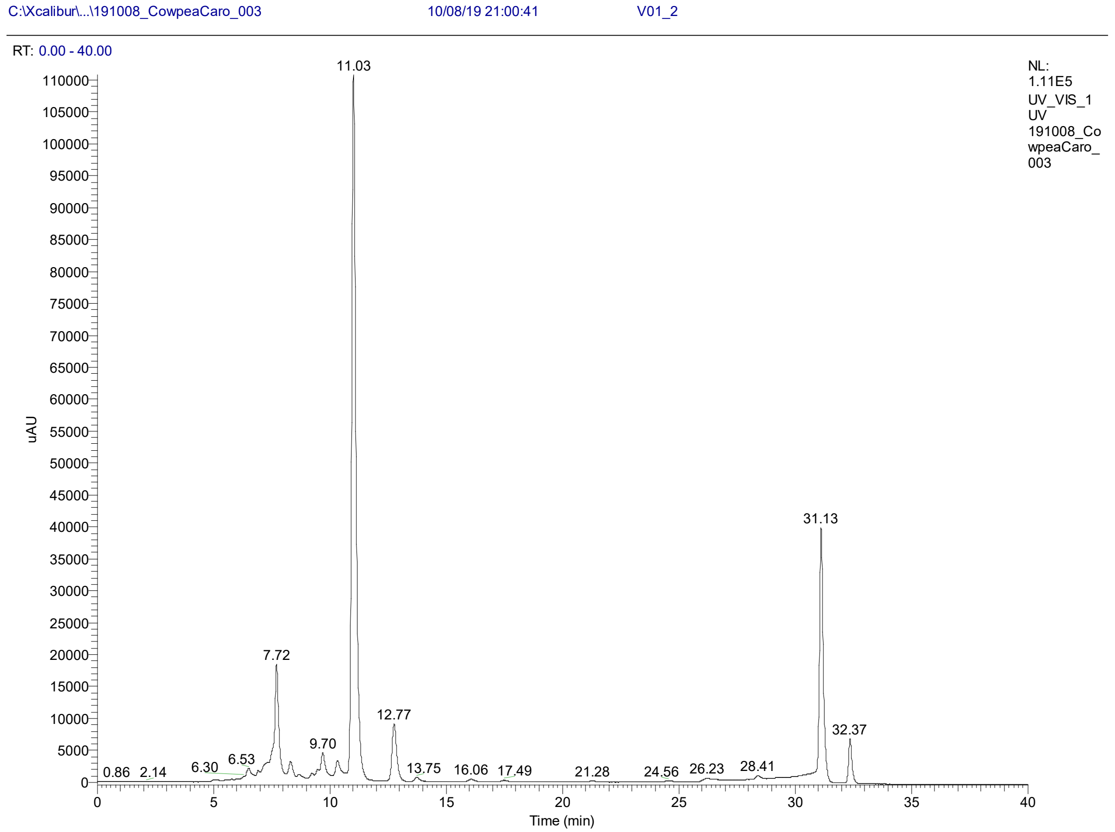

Supplementary Figure 6A: Representative UV chromatogram of carotenoids in cowpea leaves at @450 nm

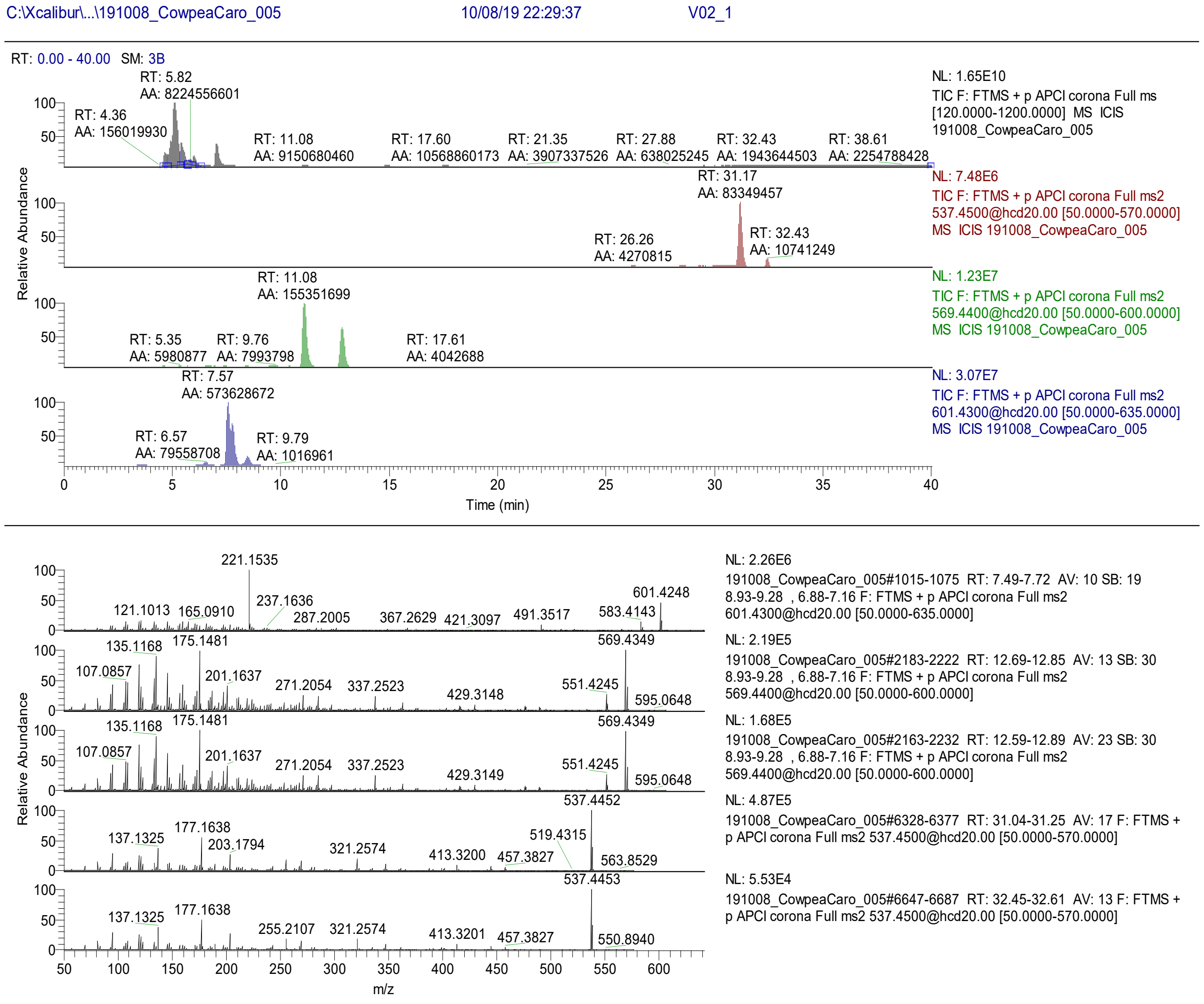

Supplementary Figure 6B: Representative TIC and mass features of individual carotenoid compounds detected in cowpea leaves

Supplementary Table 1. Primer sequences used to amplify, the GLUT4 and GAPDH cDNA

Gene	Primer	PCR product size
GLUT 4	Forward: ACATACCTGACAGGGCAAGG<br>Reverse: CGCCCTTAGTTGGTCAGAAG	152bp
GAPDH (Housekeeping genes)	Forward: ACTTTGGCATTGTGGAAGG<br>Reverse: ACACATTGGGGGTAGGAACA	223bp

Seabi *et al*. [18]

Supplementary Table 2. Pearson's correlation coefficients between targeted phenolic components and *in vitro* antioxidant (FRAP), α-glucosidase and α-amylase activities

Targeted phenolic components	FRAP activity	α-glucosidase	α-amylase activity
**Gentisic acid-5-O-glucoside**	0.75	0.78	0.70
Coumaric acid-O-glucoside	0.69	0.58	0.59
Ferulic acid-O-glucoside	0.65	0.60	0.64
**Quercetin 3-(2G-xylosylrutinoside)**	0.70	0.69	0.67
**Quercetin 3-glucosyl-(1->2)-galactoside,**	0.72	0.75	0.65
**Quercetin 3-O-rhamnoside 7-O-glucoside**	0.57	0.42	0.48
**Quercetin 3-sambubioside-3'-glucoside**	0.70	0.63	0.60

Supplementary Table 3 Characterization of carotenoid compounds detected in cowpea accessions by LC–APCI-MS scanning at positive mode.

Tentative identification	Retention time min	Molecular ion [M-H]+	Empirical molecular formula	Fragments	UV max (nm)	References
Violaxanthin	6.53/7.72/8.47	601.4251	$C_{46}H_{57}O_4^+$	583.4143, 491.3517, 221.1535	415/439/469	[41]
Lutein	11.03	569.4349	$C_{40}H_{57}O_2^+$	551.4245, 476.3646, 431.3306, 337.2523, 175.1481	446/474	
Zeaxanthin	12.77	569.4355	$C_{40}H_{57}O_2^+$	551.4246, 476.3647, 337.2523, 175.1482	449/476	
All-trans-beta-carotene	31.13	537.4455	$C_{40}H_{57}^+$	481.3826, 413.3201, 177.1852, 137.1325	452/478	
9-cis-beta-carotene	32.37	537.4455	$C_{40}H_{57}^+$	413.3201, 321.2575, 177.1851, 137.1325	445/473	
